# Normal oil body formation in *Marchantia polymorpha* requires functional coat protein complex I proteins

**DOI:** 10.3389/fpls.2022.979066

**Published:** 2022-08-15

**Authors:** Takehiko Kanazawa, Ryuichi Nishihama, Takashi Ueda

**Affiliations:** ^1^Division of Cellular Dynamics, National Institute for Basic Biology, Okazaki, Aichi, Japan; ^2^The Department of Basic Biology, SOKENDAI (The Graduate University for Advanced Studies), Okazaki, Aichi, Japan; ^3^Department of Applied Biological Science, Faculty of Science and Technology, Tokyo University of Science, Noda, Japan

**Keywords:** *Marchantia polymorpha*, oil body, membrane traffic, Golgi apparatus, secretory pathway, COPI coat

## Abstract

Eukaryotic cells possess endomembrane organelles equipped with specific sets of proteins, lipids, and polysaccharides that are fundamental for realizing each organelle’s specific function and shape. A tightly regulated membrane trafficking system mediates the transportation and localization of these substances. Generally, the secretory/exocytic pathway is responsible for transporting cargo to the plasma membrane and/or the extracellular space. However, in the case of oil body cells in the liverwort *Marchantia polymorpha,* the oil body, a liverwort-unique organelle, is thought to be formed by secretory vesicle fusion through redirection of the secretory pathway inside the cell. Although their formation mechanism remains largely unclear, oil bodies exhibit a complex and bumpy surface structure. In this study, we isolated a mutant with spherical oil bodies through visual screening of mutants with abnormally shaped oil bodies. This mutant harbored a mutation in a coat protein complex I (COPI) subunit MpSEC28, and a similar effect on oil body morphology was also detected in knockdown mutants of other COPI subunits. Fluorescently tagged MpSEC28 was localized to the periphery of the Golgi apparatus together with other subunits, suggesting that it is involved in retrograde transport from and/or in the Golgi apparatus as a component of the COPI coat. The Mp*sec28* mutants also exhibited weakened stiffness of the thalli, suggesting impaired cell–cell adhesion and cell wall integrity. These findings suggest that the mechanism of cell wall biosynthesis is also involved in shaping the oil body in *M. polymorpha*, supporting the redirection of the secretory pathway inward the cell during oil body formation.

## Introduction

The trafficking system between endomembranous organelles, including the plasma membrane (PM) in eukaryotic cells, is responsible for the precise transportation and localization of proteins, lipids, and polysaccharides, and is referred to as membrane traffic. The membrane trafficking system mainly consists of the secretory, endocytic, and lysosomal/vacuolar transport pathways. The secretory, endocytic, and lysosomal/vacuolar transport pathways mediate transport from intracellular organelles to the extracellular space or PM, the extracellular space or PM to intracellular organelles, and to the lysosomes/vacuole, respectively (Kanazawa and Ueda, 2017; Aniento et al., 2022). A single round of trafficking between two compartments is initiated by sorting cargo and forming transport vesicles on a donor organelle membrane, generally mediated by SAR1/ARF GTPases and coat protein complexes, such as coat protein complexes I and II (COPI and COPII) and the clathrin coat (Dacks and Robinson, 2017; Bethune and Wieland, 2018; Dell'Angelica and Bonifacino, 2019). RAB GTPases mediate the targeting and tethering of transport vesicles to target organelle membranes. SNARE proteins execute membrane fusion between these membranes to unload cargo molecules (Kanazawa and Ueda, 2017; Aniento et al., 2022; Ito and Uemura, 2022). Among the coat protein complexes, COPI acts in retrograde transport from the Golgi apparatus to the endoplasmic reticulum (ER) and in intra-Golgi trafficking. The COPI coat is composed of hetero-heptameric subunits α, β, β′, γ, δ, ε, and ζ, which are highly conserved among eukaryotes (Gao et al., 2014; Ito et al., 2014; Dodonova et al., 2015; Dacks and Robinson, 2017).

Although the fundamental molecular framework and major membrane trafficking pathways are highly conserved among eukaryotic lineages, some lineages have also acquired lineage-specific organelles, which should be accompanied by the development of new trafficking pathways to or from these organelles. Comparative genomics analyses have inferred the molecular mechanisms underlying the emergence of new endomembranous organelles and membrane trafficking pathways and detected lineage-specific acquisition of key machinery components, such as coat protein complexes, RAB GTPases, and SNARE proteins. Given that practically all extant membrane trafficking pathways occur through the actions of these machinery components, acquiring a novel membrane trafficking pathway should be accompanied by obtaining a new set of these components. Attaining these components with differentiated functions is thought to be accomplished through gene duplication followed by neo- and/or sub-functionalization by accumulated mutations (Dacks and Field, 2007; Schlacht et al., 2014). Co-opting evolutionarily conserved machinery components with distinct membrane trafficking pathways would be another possibility to explore new trafficking pathways. These theoretical frameworks have been proposed for many years, based on trans-lineage phylogenetic analyses. However, empirical support for these frameworks is limited.

The liverwort oil body is a striking example of a lineage-unique organelle specifically acquired by distinctive eukaryotic lineages during evolution. Notably, the liverwort oil body is an organelle independent from the oil body in seed plants (also known as the oleosome or lipid body) in regard to its origin or function; the liverwort oil body is surrounded by the phospholipid bilayer membrane similar to endomembrane organelles and probably acts in chemical protection against microbes and herbivores, whereas the seed plant oil body is surrounded by a lipid monolayer derived from the ER and accumulates neutral lipids as energy sources (Huang, 1992; He et al., 2013; Huang et al., 2013; Kanazawa et al., 2020; Romani et al., 2020, 2022). The morphology and distribution patterns of the liverwort oil body, such as the shape, color, and number in a cell, vary depending on the liverwort species and have thus been used as an index for taxonomically classifying liverworts (Müller, 1939; He et al., 2013; Romani et al., 2022). In the liverwort *Marchantia polymorpha*, one oil body is formed in a specific idioblastic cell, referred to as the oil body cell. Recent analyses have revealed that at least two key transcription factors, MpC1HDZ and MpERF13, mediate oil body cell differentiation and oil body development in *M*. *polymorpha* (Kanazawa et al., 2020; Romani et al., 2020, 2022). The Qa-SNARE protein MpSYP12B, which is homologous to the PM-resident MpSYP13B protein, is targeted to the oil-body membrane (Kanazawa et al., 2016). Artificial secretory cargo, sec-mRFP, is transported to the oil body lumen when expressed under Mp*SYP12B* promoter regulation in oil body cells, whereas sec-mRFP, driven by the Mp*SYP13B* promoter, is secreted to the extracellular space (Kanazawa et al., 2020). Alongside other supporting evidence, it was proposed that cellular state-specific redirection of the secretory pathway forms the oil body.

Genome information and a series of versatile molecular genetic tools are available for *M. polymorpha* studies (Ishizaki et al., 2016; Bowman et al., 2017; Montgomery et al., 2020; Kohchi et al., 2021; Bowman, 2022; Bowman et al., in press), including the high-efficiency *Agrobacterium*-mediated transformation of sporelings (Ishizaki et al., 2008). Using this transformation method can generate several transgenic plants in which transfer DNA (T-DNA) is randomly inserted into the genome, enabling mutants defective in gene functions involved in various biological processes, such as air chamber formation (Ishizaki et al., 2013), rhizoid development (Honkanen et al., 2016, 2018; Proust et al., 2016), lateral organ development (Naramoto et al., 2019), and oil body formation (Kanazawa et al., 2020), to be screened. In this study, to obtain further insights into oil body biogenesis, we screened a T-DNA-mutagenized population of *M*. *polymorpha* for mutants with abnormally shaped oil bodies and isolated a mutant with oil bodies with increased circularity. By analyzing the gene responsible for this mutation and other related genes, we demonstrated that normal oil body formation in *M*. *polymorpha* requires COPI-mediated secretory activity.

## Materials and methods

### Plant materials and transformation

*Marchantia polymorpha* accessions Takaragaike-1 (Tak-1, male) and Takaragaike-2 (Tak-2, female; Ishizaki et al., 2008) were used as the wild-type. Supplementary Table 1 lists the plants used in this study. The growth conditions and transformation methods have been previously described (Ishizaki et al., 2008; Kubota et al., 2013; Kanazawa et al., 2020). The thalli were grown asexually and maintained on 1/2 × Gamborg’s B5 medium containing 1.0% (w/v) agar at 22°C under continuous white light. The transition from vegetative to reproductive growth was induced by supplementation with far-red light to obtain spores by crossing male and female lines. Gemmae were cultured on 1/2 × Gamborg’s B5 medium plates containing 1.0% (w/v) sucrose at 22°C under continuous white light for 5 days, then transferred onto 1/2 × Gamborg’s B5 medium plates containing 20 μM β-estradiol (Fujifilm) or the equivalent volume [0.1% (v/v)] of DMSO (Fujifilm) as a mock control to evaluate the effect of Mp*SEC21* and Mp*RET1* knockdown on thallus development. The area of the thalli was measured using ImageJ software (version 1.53 k).^1^

### Isolating the T-DNA-insertion mutant

We screened for mutants from T-DNA-inserted transgenic plants and identified the causal genes by TAIL-PCR as previously described by Kanazawa et al. (2020). Briefly, 48,825 T-DNA-inserted plants were obtained by the co-culture of *M*. *polymorpha* sporelings with agrobacteria harboring the binary vector pCAMBIA1300, followed by selection on hygromycin-supplemented medium plates. Hygromycin-resistant plants were stained with BODIPY 493/503 (4,4-difluoro-1,3,5,7,8-pentamethyl-4-bora-3a,4a-diaza-*s*-indacene, Thermo Fisher), and mutants with abnormal oil-body morphology were examined under a fluorescent stereoscopic microscope (M165FC, Leica). The T-DNA flanking sequences were amplified by TAIL-PCR and sequenced, and the obtained sequences were subjected to a BLAST search using MarpolBase.^2^

### PCR-based genotyping

Sample preparation and PCR for genotyping were performed as described by Kanazawa et al., 2020. Supplementary Table 2 lists the primers used.

### Phylogenetic analysis

Protein sequences were collected from Phytozome^3^ and the following genome portal sites: *Amborella trichopoda* v1.0, *Arabidopsis thaliana* TAIR10, *Brachypodium distachyon* v3.1, *Chlamydomonas reinhardtii* v5.5/v5.6, *Klebsormidium nitens* NIES-2285 V1.1,^4^ *Medicago truncatula* Mt4.0v1, *Oryza sativa* v7_JGI, *Marchantia polymorpha* v5.1,^5^ *Physcomitrium patens* v3.3, *Populus trichocarpa* v3.0/v4.1, *Selaginella moellendorffii* v1.0, *Sphagnum fallax* v0.5/v1.1, and *Ostreococcus lucimarinus* v2.0. A multiple sequence alignment was performed using the MUSCLE program version 3.8.31, with default parameters (Edgar, 2004a, 2004b). Alignment gaps were removed manually or with the Gblocks program version 0.91b (Castresana, 2000; Talavera and Castresana, 2007). The maximum likelihood phylogenetic analyses were performed using PhyML 3.0 under the LG model (Guindon et al., 2010). Bootstrap analyses were performed by resampling 1,000 sets.

### Vector construction

**Figure 1:**
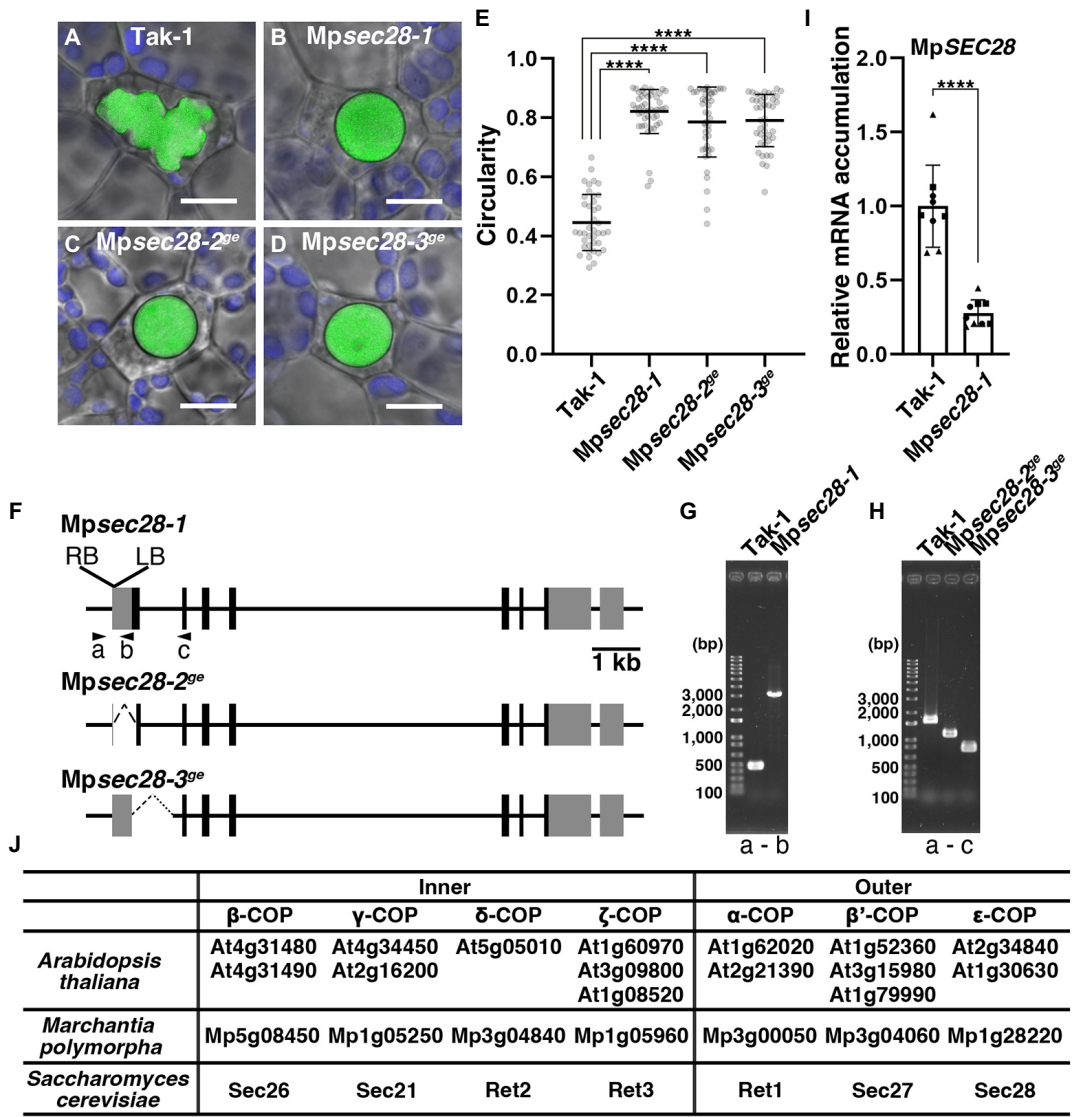
Abnormal oil body development in Mp*sec28* mutants. **(A–D)** Confocal images merged with bright-field images of oil bodies in wild-type Tak-1 **(A)**, Mp*sec28-1* **(B)**, Mp*sec28-2^ge^* **(C)**, and Mp*sec28-3^ge^* **(D)**. Green and blue pseudo-colors indicate the fluorescence from BODIPY and chlorophyll, respectively. Bars = 10 μm. **(E)** The circularity of the oil bodies in each genotype. Data are presented as the means ± s.d. Differences between wild-type and mutant plants were statistically significant based on a two-tailed Welch’s *t*-test. ****p < 0.001. The sample numbers were 40, 47, 44, and 42 oil bodies for Tak-1, Mp*sec28-1*, Mp*sec28-2^ge^*, and Mp*sec28-3^ge^*, respectively. *p* = 8.23 × 10^−32^ for Mp*sec28-1*, *p =* 2.73 × 10^−24^ for Mp*sec28-2^ge^*, and *p =* 4.40 × 10^−28^ for Mp*sec28-3^ge^*. **(F)** Schematic representation of the Mp*SEC28* gene structure and mutations generated in this study. Boxes indicate the UTRs (gray) and coding sequences (black). **(G,H)** PCR-based genotyping of Tak-1 and Mp*sec28-1* **(G)**, Tak-1, Mp*sec28-2^ge^*, and Mp*sec28-3^ge^* **(H)**. **(F)** Shows the primer **(A–C)** annealing sites. **(I)** Relative Mp*SEC28* mRNA accumulation in Tak-1 and Mp*sec28-1* measured by qRT-PCR. Mp*APT* was used as an internal reference. Data are presented as the means ± s.d. The difference between Tak-1 and Mp*sec28-1* was statistically analyzed by a two-tailed Welch’s *t*-test. ^****^p < 0.001. Three biological replicates were prepared, and experiments were performed three times for each gene. Each biological replicate is indicated by a circle (replicate 1), triangle (replicate 2), or square (replicate 3). **(J)** Genes encoding COPI complex subunits in *Arabidopsis thaliana*, *Marchantia polymorpha*, and *Saccharomyces cerevisiae*.

Supplementary Tables 2, 3 list the oligonucleotides and plasmids used in this study, respectively. Open reading frames (ORFs) and genomic sequences were amplified by PCR from complementary DNA (cDNA) and genomic DNA prepared from *M*. *polymorpha* accession Tak-1, respectively, for entry vector construction. The amplified products were subcloned into pENTR/D-TOPO (Thermo Fisher) according to the manufacturer’s instructions. mCherry cDNA with a linker sequence was inserted into the *Asc*I site of the pENTR vector containing the cDNA for Mp*SEC28* using the In-Fusion HD cloning kit (Clontech), according to the manufacturer’s instructions. The PCR-amplified DNA fragment for mCitrine was inserted into the *Sac*I site of pMpGWB301 using an In-Fusion cloning kit to construct the pMpGWB301-*mCitrine* destination vector harboring the Gateway cassette. Target sequences for genome editing were selected from the CRISPRdirect website^6^ (Naito et al., 2015). Double-stranded DNA fragments of the target sequences were subcloned into pMpGE_En04 to construct dual-gRNA vectors (Koide et al., 2020). The dual-gRNA system was used to generate mutants with a large genomic sequence deletion (Sugano et al., 2018). However, gRNA destined for the 3′-end of the Mp*SEC28* gene did not work in this study, resulting in the mutations indicated in Figure 1F. For the amiRNA experiments, Mp*MIR160* was used as the backbone according to Flores-Sandoval et al., 2016. The amiRNA sequences were amplified by PCR and subcloned into the pENTR/D-TOPO vector.

The entry clones described above were transferred to destination vectors (pMpGWB101, pMpGWB301; Ishizaki et al., 2015), pMpGWB301-*mCitrine*, or pMpGWB301*_pro_* Mp*SYP2*:GW-*mCitrine* for expression of chimeric proteins with fluorescent proteins, pMpGE011 for genome editing (Sugano et al., 2018), and pMpGWB368 (Furuya et al., 2022; Ishida et al., 2022) for β-estradiol-induced amiRNA expression using Gateway LR Clonase II Enzyme Mix (Thermo Fisher) according to the manufacturer’s instructions.

### Confocal laser scanning microscopy and image analyses

Five-day-old thalli were used for observation. For BODIPY staining, thalli were incubated in 200 nM BODIPY 493/503, dissolved in water for 30 min, washed twice in water, and used for microscopic observation. For β-estradiol treatment, gemmae were grown on a 1/2 × Gamborg’s B5 medium plate for 3 days, then soaked in a liquid medium containing 20 μM β-estradiol or 0.1% (v/v) DMSO for 48 h. The samples were mounted in a 1/2 × Gamborg’s B5 liquid medium and observed using an LSM780 confocal microscope (Carl Zeiss) equipped with an oil immersion lens (63 ×, numerical aperture = 1.4) and lambda and Airyscan detectors. For spectral imaging, the samples were excited at 488 nm (Argon 488) and 561 nm (DPSS 561–10), and emissions between 482 and 659 nm were collected. For high-resolution imaging using the Airyscan detector, samples were excited at 488 and 561 nm, and the emission was separated using a BP495-550 + BP570-630 filter. The images were acquired using line scanning. Spectral unmixing and Airy processing of the obtained images were performed using ZEN2.3 SP1 software (Carl Zeiss). We calculated the circularity of the oil bodies and analyzed colocalization using ImageJ and the Coloc_2 plugin in ImageJ Fiji (Schindelin et al., 2012), respectively.

### Quantitative reverse transcription (qRT)-PCR

The β-estradiol treatment was performed as described in the microscopy section. Total RNA was extracted as described by Kanazawa et al., 2013 and Kanazawa et al., 2020. cDNA was synthesized from total RNA using oligo dT(18) primers and SuperScript III Reverse Transcriptase (Thermo Fisher) according to the manufacturer’s instructions. qRT-PCR was performed with a LightCycler 96 (Roche) using the KAPA SYBR Fast qPCR Master Mix Kit (KAPA Biosystems) according to the manufacturer’s protocol. Mp*APT* was used as the housekeeping reference for normalization (Saint-Marcoux et al., 2015). Three biological replicates were prepared, and each experiment was repeated two or three times. Semi-quantitative RT-PCR was performed using KOD FX Neo (Toyobo), according to the manufacturer’s instructions.

### Statistics and reproducibility

We present representative confocal microscopy images of biological and technical replicates. The Source data list the results of quantitative image analyses and qRT-PCR, the number of generated transgenic lines, and the number of observed samples. Statistical analyses were performed using the Excel 365 software (ver. 2,202) or GraphPad Prism 9.3.1. (GraphPad Software), and graphs were drawn using GraphPad Prism 9.3.1.

### Semi-quantitative chopping assay

Scheme of the assay is shown in Supplementary Figure 9. Twelve-day-old thalli were placed on 5 mm-gapped blocks. The blocks were placed on an electronic force balance, and the thalli were pressed using tweezers to record the force that severed the thalli.

## Results

### Isolating a T-DNA-inserted mutant with abnormally shaped oil bodies

The oil bodies of *M*. *polymorpha* can be visualized by staining with the lipophilic dye BODIPY 493/503 (Kanazawa et al., 2020). This allowed us to screen mutants with aberrantly shaped oil bodies. One of these mutants, Mp*sec28-1* (identification of the responsible gene is described below), exhibited rounded oil body morphology. Oil bodies in wild-type plants exhibited a bumpy and complex morphology, with a mean circularity of 0.445 ± 0.0937 (mean ± s.d.; Figures 1A,E). The circularity of the oil body in the Mp*sec28-1* mutant was 0.821 ± 0.0740, which was significantly higher than that of the wild-type (Figures 1B,E). This phenotype was heritable. The abnormal oil body phenotype was detected in 109 of 224 F1 plants obtained by crossing the Mp*sec28-1* mutant with wild-type plants (*p* = 0.6885 in *χ*-squared test). This oil body phenotype was linked to the hygromycin resistance conferred by the *HPT* gene in the T-DNA, indicating that the phenotype was caused by a single nuclear mutation resulting from T-DNA insertion.

We performed a thermal asymmetric interlaced (TAIL)-polymerase chain reaction (PCR), sequenced the obtained DNA fragments, and conducted a BLAST search against the *M*. *polymorpha* genome sequence to identify the T-DNA insertion site. T-DNA was inserted into the 5′ untranslated region (UTR) of Mp1g28220 (Figures 1F,G). Mp1g28220 is annotated as the gene encoding the epsilon COPI subunit orthologous to Sec28 and ε-COP in *Saccharomyces cerevisiae* and mammals, respectively, which was supported by our maximum likelihood phylogenetic analysis (Figure 1J; Supplementary Figure 1; Hara-Kuge et al., 1994; Duden et al., 1998). Thus, we named this gene Mp*SEC28*. The Mp*sec28-1* allele was likely a knockdown allele, because mRNA accumulation was detected by qRT-PCR at a significantly reduced level compared to that in wild-type (Figure 1I). We generated other mutant alleles (Mp*sec28-2^ge^* and Mp*sec28-3^ge^*) using CRISPR-based genome editing, which should be knockout mutants because the start codon was deleted in these mutants (Figures 1F,H), to confirm that a mutation in Mp*SEC28* caused the abnormally shaped oil body phenotype. The mean circularities of the oil bodies in Mp*sec28-2^ge^* and Mp*sec28-3^ge^* were 0.785 ± 0.1173 and 0.790 ± 0.0875, respectively, similar to that of Mp*sec28-1* (Figure 1E). These results indicated that normal oil body formation requires Mp*SEC28*.

### Defects in COPI function result in abnormally shaped oil bodies

**Figure 2:**
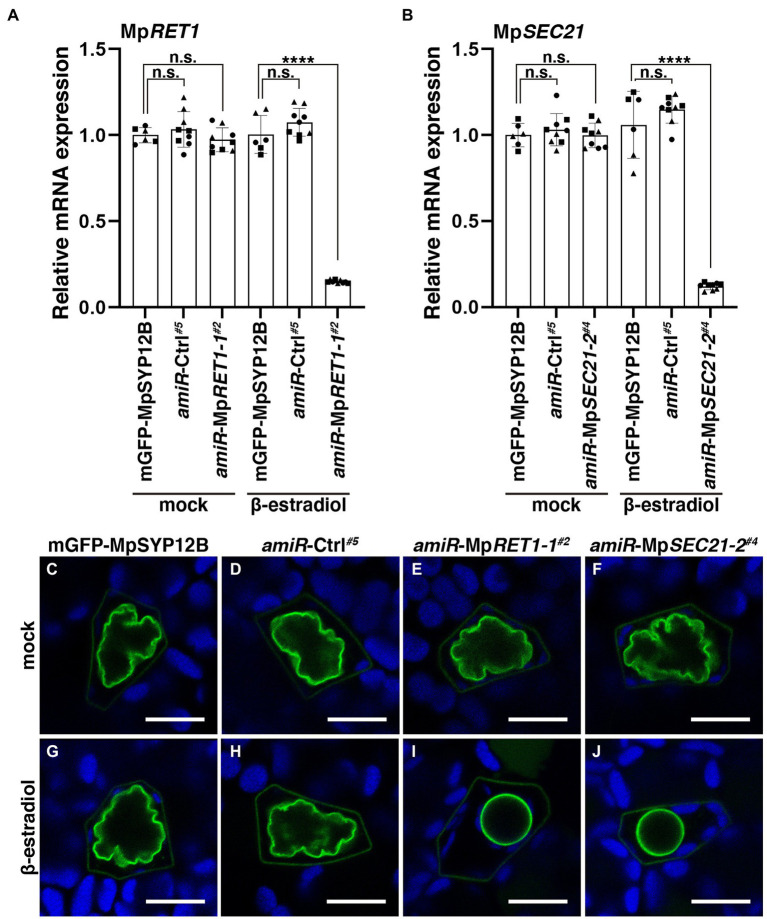
Defects in COPI function result in abnormally shaped oil bodies. **(A,B)** Relative Mp*RET1* **(A)** and MpSEC21 **(B)** mRNA expression levels in transgenic *M*. *polymorpha* plants with indicated transgenes measured by qRT-PCR. Mp*APT* was used as an internal reference. Data are presented as the means ± s.d. Significant differences between mGFP-MpSYP12B and each genotype were statistically analyzed by a two-tailed Welch’s *t*-test. n.s.: not significant; ^****^p < 0.001. Three biological replicates were prepared, and experiments were performed two or three times for each gene. Each biological replicate is indicated by a circle (replicate 1), triangle (replicate 2), or square (replicate 3). **(C–J)** Single confocal images of *M*. *polymorpha* thallus oil body cells in mGFP-MpSYP12B **(C,G)**, *amiR*-Ctrl*^#5^* **(D,H)**, *amiR*-Mp*RET1-1^#2^* **(E,I)**, and *amiR*-Mp*SEC21-2^#4^* **(F,J)** plants. Thalli were observed after 48 h of incubation with DMSO (0.1% v/v) **(C–F)** or 20 μM β-estradiol **(G–J)**. Green and blue pseudo-colors indicate the fluorescence from mGFP and chlorophyll, respectively. Bars = 10 μm. Representative images are shown among replicates. The Source data lists the number of observed cells.

The COPI coat consists of α, β, β′, γ, δ, ε, and ζ subunits and mediates intra-Golgi transport and retrograde transport from the *cis*-Golgi to the ER (Figure 1J; Glick and Nakano, 2009; Ito et al., 2014; Aniento et al., 2022). We then examined whether the oil body phenotype was specifically caused by defective MpSEC28 function or whether defective functions of other COPI subunits exerted a similar effect. In *S*. *cerevisiae*, six genes encoding COPI subunits other than *SEC28* are essential for viability (Hosobuchi et al., 1992; Duden et al., 1994, 1998; Letourneur et al., 1994; Cosson et al., 1996), which could also be the case for *M. polymorpha* COPI subunits. Therefore, we generated β-estradiol-inducible knockdown mutants of the α- and γ-subunits of the COPI-coat complex using artificial microRNA (amiRNA) for Mp*RET1* and Mp*SEC21*, respectively (Supplementary Figures 2, 3). The constructs XVE > > *amiR*-Mp*RET1-1*, XVE> > *amiR*-Mp*RET1-2*, XVE> > *amiR*-Mp*SEC21-1*, or XVE> > *amiR*-Mp*SEC21-2* were introduced into plants expressing mGFP-MpSYP12B, an oil-body membrane marker. We also introduced XVE > > *amiR*-Ctrl, in which the target sequence for Mp*RET1* or Mp*SEC21* was replaced with recognition sequences of *Sma*I and *Hin*dIII, into the mGFP-MpSYP12B plant as a negative control (Supplementary Figure 3C). After XVE > > *amiR*-Mp*RET1* and XVE > > *amiR*-Mp*SEC21* plants were treated with β-estradiol for 48 h, relative Mp*RET1* and Mp*SEC21* mRNA levels were significantly reduced compared to those in the mGFP-MpSYP12B plant (Figures 2A,B; Supplementary Figures 4A,B). In the knockdown plants, abnormally shaped oil bodies were frequently observed (Figures 2I,J; Supplementary Figure 4). A decrease in mRNA levels and abnormally shaped oil bodies upon β-estradiol treatment were not observed in the background and XVE > > *amiR*-Ctrl plants, nor in dimethyl sulfoxide (DMSO; mock)-treated XVE > > *amiR*-Mp*RET1* and XVE > > *amiR*-Mp*SEC21* plants (Figure 2; Supplementary Figure 4). These results strongly suggest that the abnormal oil body phenotype can be attributed to defective COPI function.

**Figure 3:**
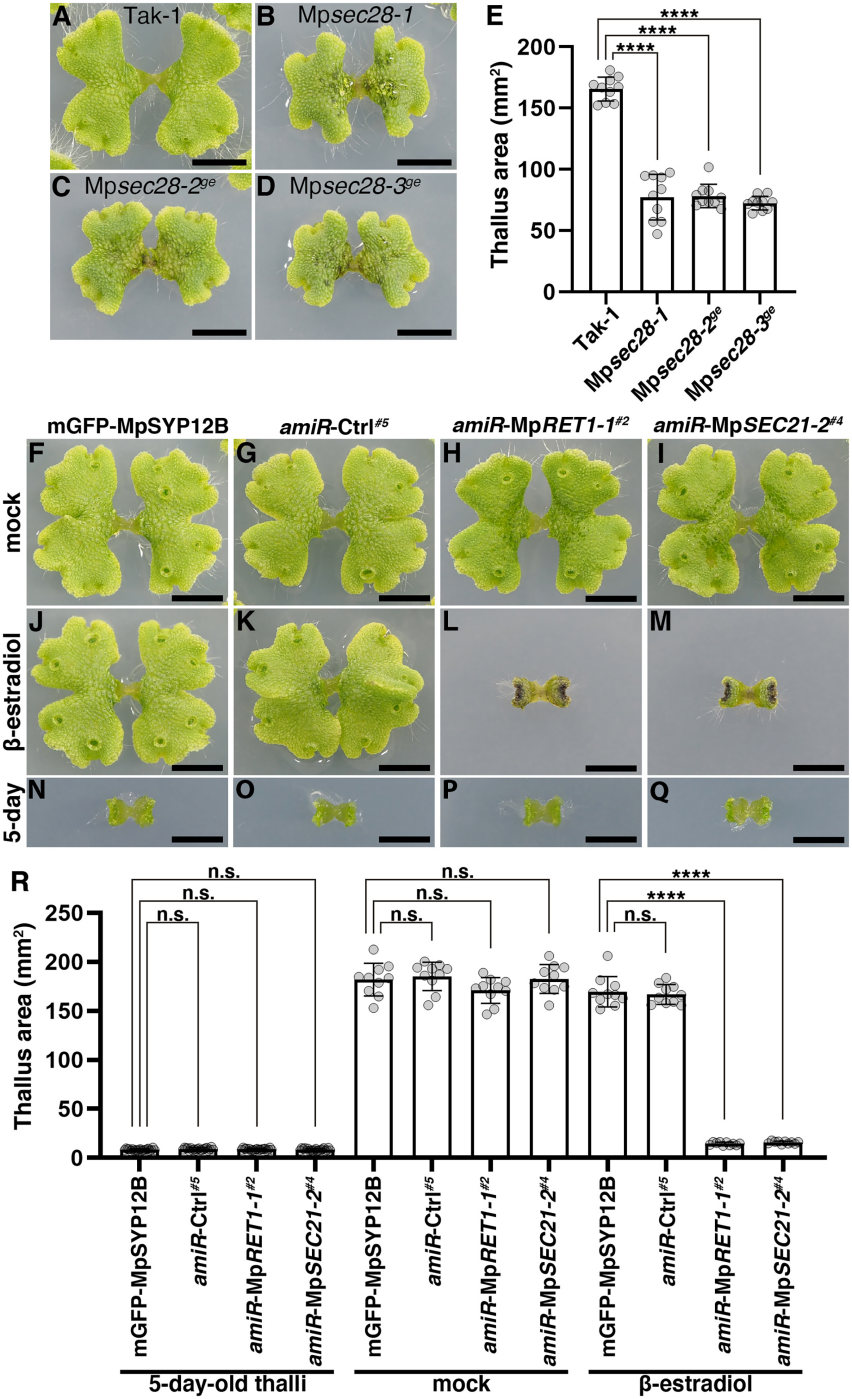
Effects of impaired COPI function on thallus growth. **(A–D)** Twelve-day-old *M*. *polymorpha* thalli of Tak-1 **(A)**, Mp*sec28-1* **(B)**, Mp*sec28-2^ge^* **(C)**, and Mp*sec28-3^ge^* **(D)**. Bars = 5 mm. **(E)** Thallus areas of plants of the indicated genotypes. Data are presented as the means ± s.d. Significant differences between Tak-1 and each mutant were statistically analyzed by a two-tailed Welch’s *t*-test. ^****^p < 0.001. Ten thalli were analyzed for each genotype. *p* = 3.64 × 10^−09^ (Mp*sec28-1*), *p =* 8.78 × 10^−14^ (Mp*sec28-2^ge^*), and *p =* 1.85 × 10^−13^ (Mp*sec28-3^ge^*). **(F–M)** *M*. *polymorpha* thalli of mGFP-MpSYP12B **(F,J,N)**, *amiR*-Ctrl*^#5^* **(G,K,O)**, *amiR*-Mp*RET1-1^#2^* **(H,L,P)**, and *amiR*-Mp*SEC21-2^#4^* **(I,M,Q)** plants. Five-day-old thalli **(N–Q)** were transferred and grown on medium plates containing 0.1% (v/v) DMSO **(F–I)** or 20 μM β-estradiol **(J–M)** for 7 days. Bars = 5 mm. **(R)** The thallus area of 5-day-old plants with indicated genotypes (left) and plants treated with DMSO (mock, middle) or β-estradiol (right) for an additional 7 days. Data are presented as the means ± s.d. Significant differences between mGFP-MpSYP12B and each of the amiR plants were statistically analyzed using a two-tailed Welch’s *t*-test. n.s., not significant; ^****^p < 0.001. Twenty (5-day-old thalli) or ten (mock and β-estradiol) thalli were analyzed.

We then examined the effects of impairments in the function of COPI components on thallus growth. The thalli of Mp*sec28* mutants were viable but were smaller than those of the wild-type (Figures 3A–E). Mp*RET1* and Mp*SEC21* knockdown conferred severe effects. Five-day-old thalli of XVE > > *amiR*-Mp*RET1* and XVE > > *amiR*-Mp*SEC21* plants grown on the normal medium exhibited no detectable abnormalities in thallus development. However, following 7 days of incubation on the medium containing β-estradiol, these plants showed severely impaired growth, whereas mock treatment did not affect thallus growth (Figures 3F–R and Supplementary Figure 5). The more severe effects of impairments in Mp*RET1* and Mp*SEC21* functions compared to the loss of function of Mp*SEC28* could reflect a difference in essentiality between these genes, as shown for orthologs in *S*. *cerevisiae* (Hosobuchi et al., 1992; Letourneur et al., 1994; Gerich et al., 1995; Duden et al., 1998; Ishii et al., 2016).

### Subcellular MpSEC28-mCherry localization in thallus cells

**Figure 4:**
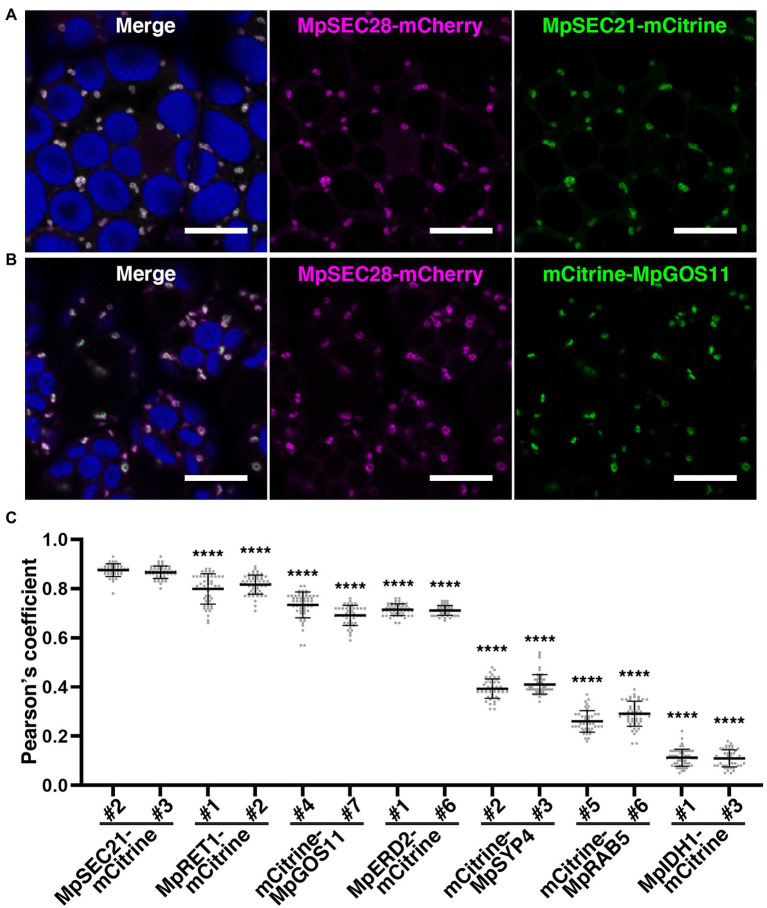
MpSEC28-mCherry colocalization with COPI components. **(A–B)** Single confocal images of *M*. *polymorpha* thallus cells expressing MpSEC28-mCherry and MpSEC21-mCitrine **(A)** or mCitrine-MpGOS11 **(B)**. Magenta, green, and blue pseudo-colors indicate fluorescence from mCherry, mCitrine, and chlorophyll, respectively. Bars = 10 μm. **(C)** Pearson correlation coefficients between the fluorescence from MpSEC28-mCherry and organelle markers. Data are presented as the means ± s.d. Significant differences between MpSEC21-mCitrine*^#2^* and each organelle marker were statistically analyzed using a two-tailed Welch’s *t*-test. ^****^p < 0.001. *n* = 50 and 51, and *p* = 2.33 × 10^−11^ and 2.25 × 10^−13^ for MpRET1-mCitrine#1 and #2, respectively. *n* = 52 and 44 and *p* = 6.71 × 10^−28^ and 6.80 × 10^−38^ for mCitrine-MpGOS11#4 and #7, respectively. *n* = 47 and 46 and *p* = 6.52 × 10^−47^ and 3.74 × 10^−47^ for MpERD2-mCitrine#1 and #6, respectively. *n* = 48 and 56 and *p* = 2.60 × 10^−74^ and 3.96 × 10^−83^ for mCitrine-MpSYP4#2 and #3, respectively. *n* = 45 and 50 and *p* = 7.07 × 10^−73^ and 5.64 × 10^−71^ for mCitrine-MpRAB5#5 and #6, respectively. *n* = 51 and 46 and *p* = 8.70 × 10^−102^ and 7.25 × 10^−94^ for MpIDH1-mCitrine#1 and #3, respectively.

**Figure 5:**
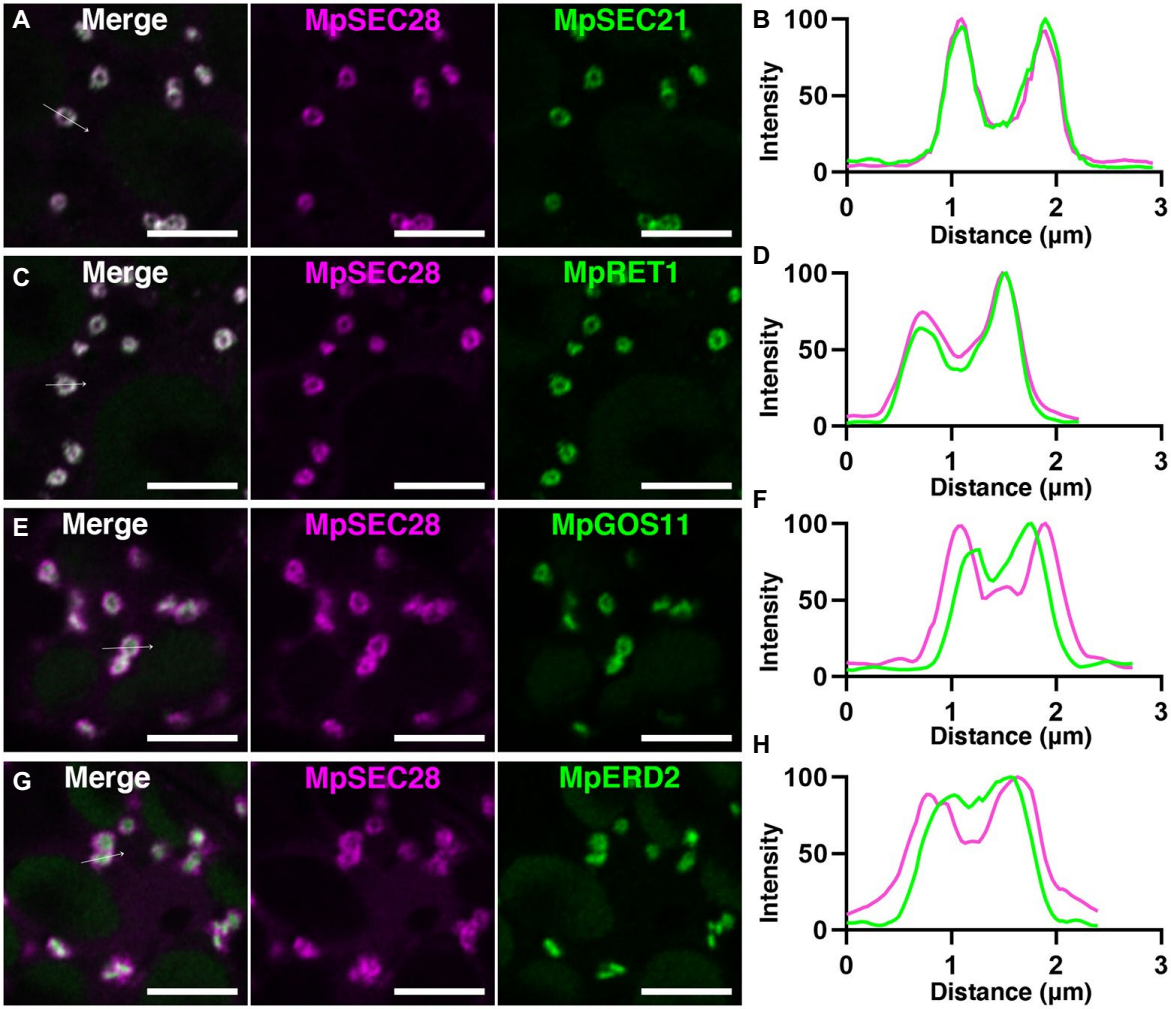
Golgi-periphery localization of MpSEC28-mCherry. **(A,C,E,G)** High spatial resolution images of *M*. *polymorpha* thallus cells expressing MpSEC28-mCherry and MpSEC21-mCitrine **(A)**, MpRET1-mCitrine **(C)**, mCitrine-MpGOS11 **(E)**, or MpERD2-mCitrine **(G)**. Magenta and green pseudo-colors indicate fluorescence from mCherry and mCitrine, respectively. Autofluorescence from chlorophyll in chloroplasts was also detected in the channel of mCitrine. Bars = 5 μm. **(B,D,F,H)** Line graphs showing relative fluorescence intensities from mCitrine and mCherry along the white arrows in A **(B)**, C **(D)**, E **(F)**, and G **(H)**.

Immunoelectron microscopy using anti-γ-COP, anti-δ-COP, and anti-ε-COP antibodies revealed that these COPI subunits are localized to vesicles close to the Golgi apparatus in root cells of Arabidopsis and maize (Pimpl et al., 2000). We generated transgenic plants expressing mCherry-tagged MpSEC28 under the regulation of its own promoter to investigate the subcellular localization of MpSEC28 in *M*. *polymorpha* and observed its subcellular localization using a confocal laser scanning microscope equipped with lambda scan or Airyscan units for spectral imaging and high-resolution imaging, respectively. MpSEC28-mCherry was observed as a ring-shaped structure with faint dispersal localization in the cytosol of thallus non-oil body cells (Figure 4). MpSEC28-mCherry was also observed as ring-like structures without any detectable localization to the oil-body membrane in oil body cells (Supplementary Figure 6A). We compared its localization with that of other fluorescent markers [MpSEC21-mCitrine (COPI-coated vesicles), MpRET1-mCitrine (COPI-coated vesicles), mCitrine-MpGOS11 (Golgi apparatus), MpERD2-mCitrine (Golgi apparatus), mCitrine-MpSYP4 [*trans*-Golgi network (TGN)], mCitrine-MpRAB5 [multivesicular body (MVB)], and MpIDH1-mCitrine (mitochondria)] to help identify the MpSEC28-mCherry-positive structure (Kanazawa et al., 2016, 2020; Minamino et al., 2017; Norizuki et al., 2022). The data obtained using the lambda scan unit were analyzed for colocalization and indicated that MpSEC28-mCherry was highly colocalized with the COPI markers MpSEC21-mCitrine and MpRET1-mCitrine (Figures 4A,C; Supplementary Figure 7A). In contrast, Pearson colocalization coefficients with MpSEC28-mCherry and mCitrine-MpGOS11, MpERD2-mCitrine, mCitrine-MpSYP4, mCitrine-MpRAB5, or MpIDH1-mCitrine were significantly lower than that with MpSEC21-mCitrine (Figures 4B,C; Supplementary Figures 7B–E). Observations with the Airyscan unit also supported the colocalization of MpSEC28-mCherry with MpSEC21-mCitrine and MpRET1-mCitrine in ring-shaped structures (Figures 5A–D), which were associated with the periphery of the Golgi apparatus labeled by mCitrine-MpGOS11 (Figures 5E,F) or MpERD2-mCitrine (Figures 5G,H). MpSEC28 did not exhibit clear colocalization with mCitrine-MpSYP4 or mCitrine-MpRAB5 (Supplementary Figure 8). These results indicate that MpSEC28 is localized to COPI-coated vesicles that form at the periphery of the Golgi apparatus in *M*. *polymorpha* thallus cells.

### Effects of Mp*sec28* mutations on thalli stiffness and transport to the oil body

**Figure 6:**
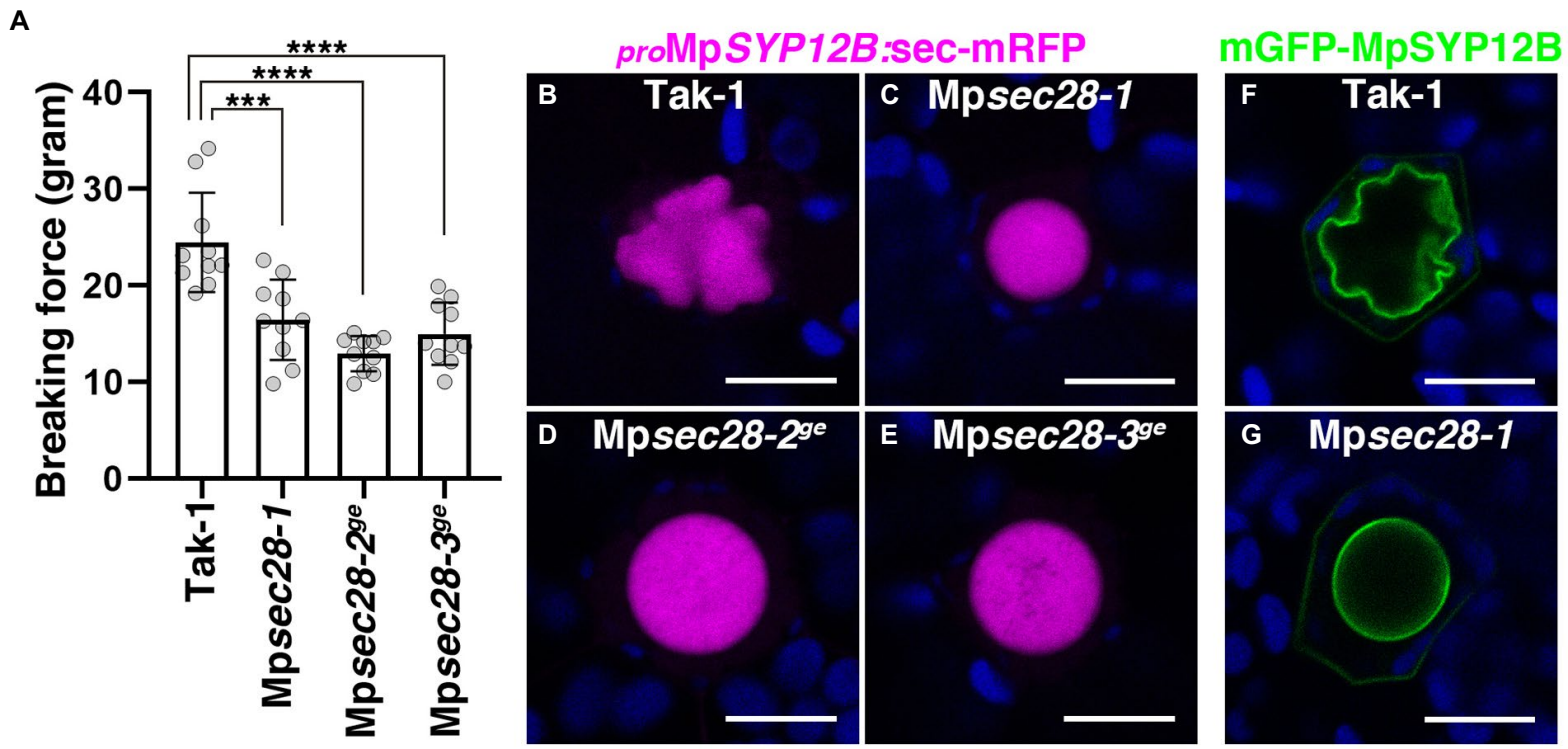
Effects of Mp*sec28* mutations on thalli stiffness and transport to the oil body. **(A)** Semi-quantitative chopping assay. The force required to sever 12-day-old thalli was measured. Data are presented as the means ± s.d. Significant differences between Tak-1 and each genotype were statistically analyzed by a two-tailed Welch’s *t*-test. ^***^p < 0.005, ^****^p < 0.001. Ten thalli were analyzed for each genotype. *p* = 1.33 × 10^−03^ for Mp*sec28-1*, *p =* 3.21 × 10^−05^ for Mp*sec28-2^ge^*, and *p =* 1.79 × 10^−04^ for Mp*sec28-3^ge^*. **(B–E)** Single confocal images of *M*. *polymorpha* thallus cells expressing sec-mRFP in Tak-1 **(B)**, Mp*sec28-1* **(C)**, Mp*sec28-2^ge^* **(D)**, and Mp*sec28-3^ge^* **(E)** under the control of the Mp*SYP12B* promoter. **(F,G)** Single confocal images of *M*. *polymorpha* thallus cells expressing mGFP-MpSYP12B under the control of its regulatory elements in Tak-1 **(F)** and Mp*sec28-1* **(G)**. Magenta, green, and blue pseudo-colors indicate fluorescence from mRFP, mGFP, and chlorophyll, respectively. Bars = 10 μm. Representative images are shown among replicates. The Source data lists the number of observed cells.

MpSEC28-mCherry and MpSEC21-mCitrine were expressed in almost all cells in five-day-old thalli, including oil body cells (Supplementary Figure 6). The mRNA levels were not significantly changed in the Mp*erf13-1^ge^* mutant, which possessed no oil body cells (Supplementary Figure 6C), indicating that Mp*SEC28* also acts in non-oil body cells. We noted that the thalli of Mp*sec28* mutants were fragile during routine handling, suggesting that Mp*sec28* mutations altered thalli stiffness. We performed a semi-quantitative chopping assay using 12-day-old thalli (Supplementary Figure 9). The mean force required to sever wild-type thalli was higher than that for Mp*sec28* mutant thalli (Figure 6A), implying that Mp*sec28* mutations affect cell wall integrity and cell–cell adhesion, probably due to the faulty secretion of substances required for cell wall integrity. Given that redirection of the secretory pathway is thought to form the oil body, transport to the oil body could also be compromised in Mp*sec28* mutants. However, we did not detect markedly affected transport of a general secretion marker sec-mRFP driven by the Mp*SYP12B* promoter or the oil-body-resident membrane protein mGFP-MpSYP12B to the oil body in Mp*sec28* mutants (Figures 6B–G). These results suggest that partially defective COPI coat function confers selective effects on secretory activity, which might be required for cell wall integrity and oil body morphogenesis in *M*. *polymorpha*.

## Discussion

The secretory pathway begins with transport from the ER to the Golgi apparatus. Most *de novo* synthesized secretory and vacuolar proteins begin their journey from the ER, mediated by COPII-coated vesicles (Barlowe et al., 1994; Bethune and Wieland, 2018). Subpopulations of ER- and Golgi-resident proteins cycle between the ER and Golgi, and COPI-coated vesicles mediate retrograde transport from the Golgi to the ER (Matsuura-Tokita et al., 2006; Glick and Nakano, 2009; Ishii et al., 2016; Nakano, 2022). The COPI coat is also responsible for the intra-Golgi transport of Golgi-resident enzymes/proteins between Golgi cisternae. In this study, we isolated the Mp*sec28-1* mutant with abnormally shaped oil bodies. MpSEC28 is orthologous to Sec28 and ε-COP, the epsilon subunits of the COPI complex in yeast and animals, respectively. The subcellular localization of MpSEC28-mCherry strongly suggested that MpSEC28 also acts as a component of the COPI coat. The mutant phenotype and responsible gene suggested that normal oil body formation requires COPI coat functionality. This was further supported by the knockdown of other putative COPI subunits, Mp*RET1* (α subunit) and Mp*SEC21* (γ subunit), which resulted in aberrant oil body morphology similar to that in Mp*sec28* mutants. Intriguingly, transport of sec-mRFP and mGFP-MpSYP12B was not markedly affected by Mp*sec28* mutations. This limited effect of mutations in Mp*SEC28* on transport of these cargos could reflect the limited requirement of MpSEC28 protein for the total secretory activity in *M. polymorpha*, which is consistent to what is known in yeast; Sec28 is an only subunit that is dispensable for viability among COPI subunits (Duden et al., 1998). Consistently, knockdown mutants of Mp*ret1* and Mp*sec21* exhibited severer developmental inhibition than Mp*sec28* mutants. Whereas the shared effect on the oil body morphology among mutations in COPI subunit genes suggests that the COPI activity is critical for normal oil body formation, we do not rule out the possibility that the severe inhibition of plant growth indirectly affected the oil body development when Mp*RET1* or Mp*SEC21* was knocked down.

It is interesting to ask why the mutants defective in COPI function exhibited abnormal oil body morphology. Mechanical support is required to shape and maintain the textured surface of the oil body in *M. polymorpha*. Given that the oil body lumen should be topologically equivalent to the extracellular space (Kanazawa et al., 2020), a cell wall-like structure might line the luminal side of the oil-body membrane, providing mechanical support to the oil body surface. Mutations in Mp*SEC28* resulted in thalli fragility suggesting that cell wall integrity requires COPI-mediated exocytic trafficking. The rounder shape of the oil body in mutants defective in COPI function could be due to the compromised integrity of the lining of the oil body lumen.

Arf GTPase activation triggers COPI coat assembly in yeast and animal cells and is catalyzed by Arf guanine nucleotide exchange factors (Arf GEFs; Stearns et al., 1990; Serafini et al., 1991; Waters et al., 1991; Donaldson et al., 1992; Hara-Kuge et al., 1994; Peyroche et al., 1996; Donaldson and Jackson, 2011; Nielsen, 2020). In Arabidopsis, two Arf GEFs with partially redundant functions, GNOM and GNL1, have been shown to act in the early secretory pathway by regulating COPI-coated vesicle formation (Richter et al., 2007; Teh and Moore, 2007; Naramoto et al., 2014; Singh et al., 2018). Calli of a mutant *GNOM* allele (*emb30*) exhibit defective cell–cell adhesion and increased fragility (Shevell et al., 2000), suggesting that cell–cell adhesion requires COPI-mediated trafficking in Arabidopsis. This is not surprising, given that the Golgi apparatus in plants is the site for pectic polysaccharide biosynthesis, which are crucial in cell–cell adhesion and the mechanical stiffness of plant tissues (Atmodjo et al., 2013; Hoffmann et al., 2021; Codjoe et al., 2022). In *M. polymorpha*, these Golgi apparatus functions should be conserved, which could account for the fragile nature of Mp*sec28* mutants. Notably, major secretory activity, including the transport of PM proteins and general secretion, is redirected toward the oil body during oil body formation in oil body cells (Kanazawa et al., 2020). In such situations, the transport of cell wall-related enzymes and polysaccharides could also be redirected toward the oil body, accommodating the polysaccharide lining with the oil-body membrane to confer mechanical strength for the formation and/or maintenance of the complex and bumpy shape of the oil body. Verifying this possibility and unraveling the precise role of the COPI coat in oil body formation would be an interesting future project.

*SEC28*/*ε-COP* knockdown causes mislocalization of Golgi-resident Arabidopsis endomembrane protein 12 (AtEMP12), which harbors the KXD/E motif that mediates the interaction with the COPI coat at the C-terminal cytosolic tail (Woo et al., 2015). In this plant, the number of cisternae in the Golgi stack is significantly decreased compared to the Golgi in wild-type plants, suggesting that COPI is crucial in maintaining Golgi functions in plant cells. In our previous study, we proposed that the oil body is formed through redirection of the secretory pathway inward of the cell (Kanazawa et al., 2020), but the involvement of the Golgi apparatus and machinery components of membrane traffic in oil body formation remains elusive. In this study, we showed that the COPI coat is pivotal in normal oil body formation, strongly suggesting that the Golgi-dependent transport pathway substantially contributes to oil body formation. The results obtained in this study further support the proposal that the redirection of the secretory pathway forms the oil body in *M. polymorpha*. However, it is still possible that other subcellular activities dependent on the ER-Golgi trafficking are also involved in oil body formation. Further studies are needed to elucidate precise molecular and regulatory mechanisms of oil body formation in liverworts.

## Data availability statement

The original contributions presented in the study are included in the article/Supplementary material, further inquiries can be directed to the corresponding author.

## Author contributions

TK performed the experiments and data analyses. RN designed and constructed vectors. TU supervised the study. TK and TU wrote the manuscript. All authors contributed to the article and approved the submitted version.

## Funding

This work was financially supported by Grants-in-Aid for Scientific Research from the Ministry of Education, Culture, Sports, Science, and Technology of Japan [19H05670, 19H05675, 21H02515 (TU), and 22K06272 (TK)].

## Conflict of interest

The authors declare that the research was conducted in the absence of any commercial or financial relationships that could be construed as a potential conflict of interest.

## Publisher’s note

All claims expressed in this article are solely those of the authors and do not necessarily represent those of their affiliated organizations, or those of the publisher, the editors and the reviewers. Any product that may be evaluated in this article, or claim that may be made by its manufacturer, is not guaranteed or endorsed by the publisher.

## Footnotes

^1^ http://imagej.nih.gov/ij

^2^ https://marchantia.info/

^3^ https://phytozome-next.jgi.doe.gov/

^4^ http://www.plantmorphogenesis.bio.titech.ac.jp/~algae_genome_project/klebsormidium/

^5^ http://marchantia.info/

^6^ https://crispr.dbcls.jp/
