# Supplementary material for: Normal oil body formation in Marchantia polymorpha requires functional coat protein complex I proteins
Source: Front Plant Sci. 2022 Aug 15;13:979066. doi: 10.3389/fpls.2022.979066 (PMC9420845; doi:10.3389/fpls.2022.979066)
Supplement: Supplementary file 1 [file Data_Sheet_1.PDF]

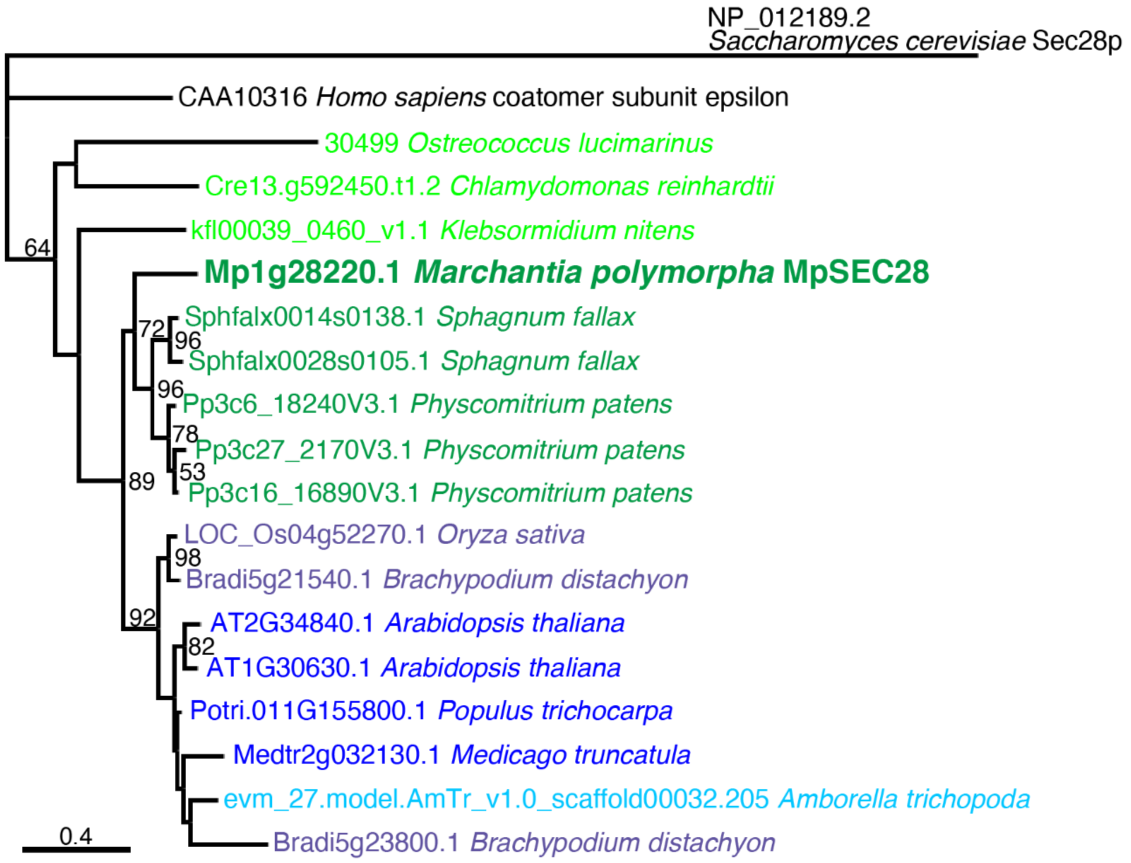

Supplementary Figure 1. A maximum likelihood phylogenetic tree of SEC28/ε-COP. *Saccharomyces cerevisiae* and *Homo sapiens* sequences were used as the outgroups. Bootstrap values over 50% are indicated. Black, light green, dark green, light blue, purple, and blue OTUs indicate the outgroup, green algae, bryophytes, basal angiosperm, monocots, and dicots, respectively. The branch lengths are proportional to the estimated number of substitutions per site.

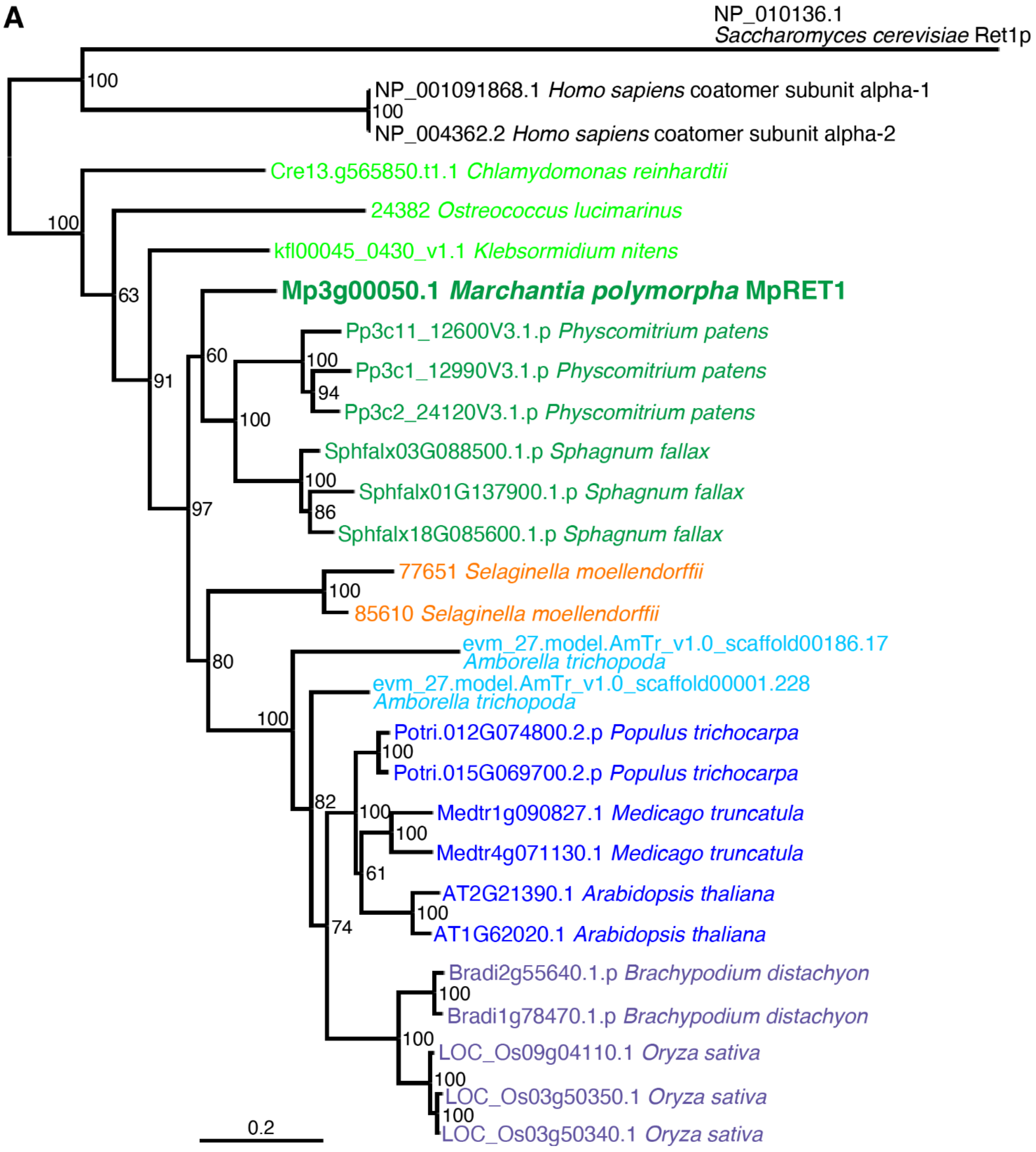

Supplementary Figure 2. Phylogenetic analyses of RET1/α-COP and SEC21/γ-COP. (A, B) Maximum likelihood phylogenetic trees of RET1/α-COP **(A)** and SEC21/γ-COP **(B)**. *Saccharomyces cerevisiae* and *Homo sapiens* sequences were used as the outgroups. Bootstrap values over 50% are indicated. Black, light green, dark green, orange, light blue, purple, and blue OTUs indicate the outgroup, green algae, bryophytes, lycophytes, basal angiosperm, monocots, and dicots, respectively. The branch lengths are proportional to the estimated number of substitutions per site.

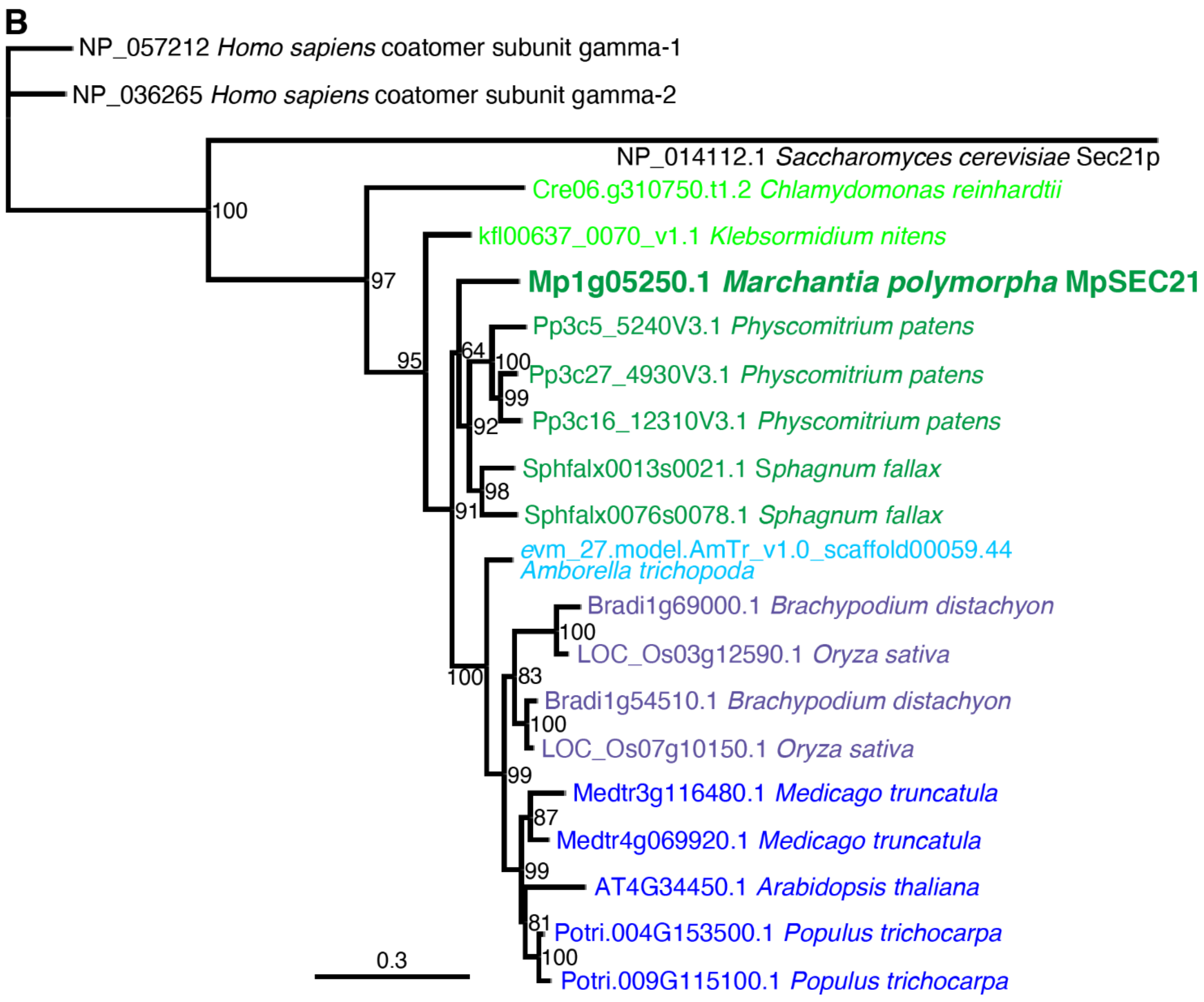

Supplementary Figure 2. (Continued).

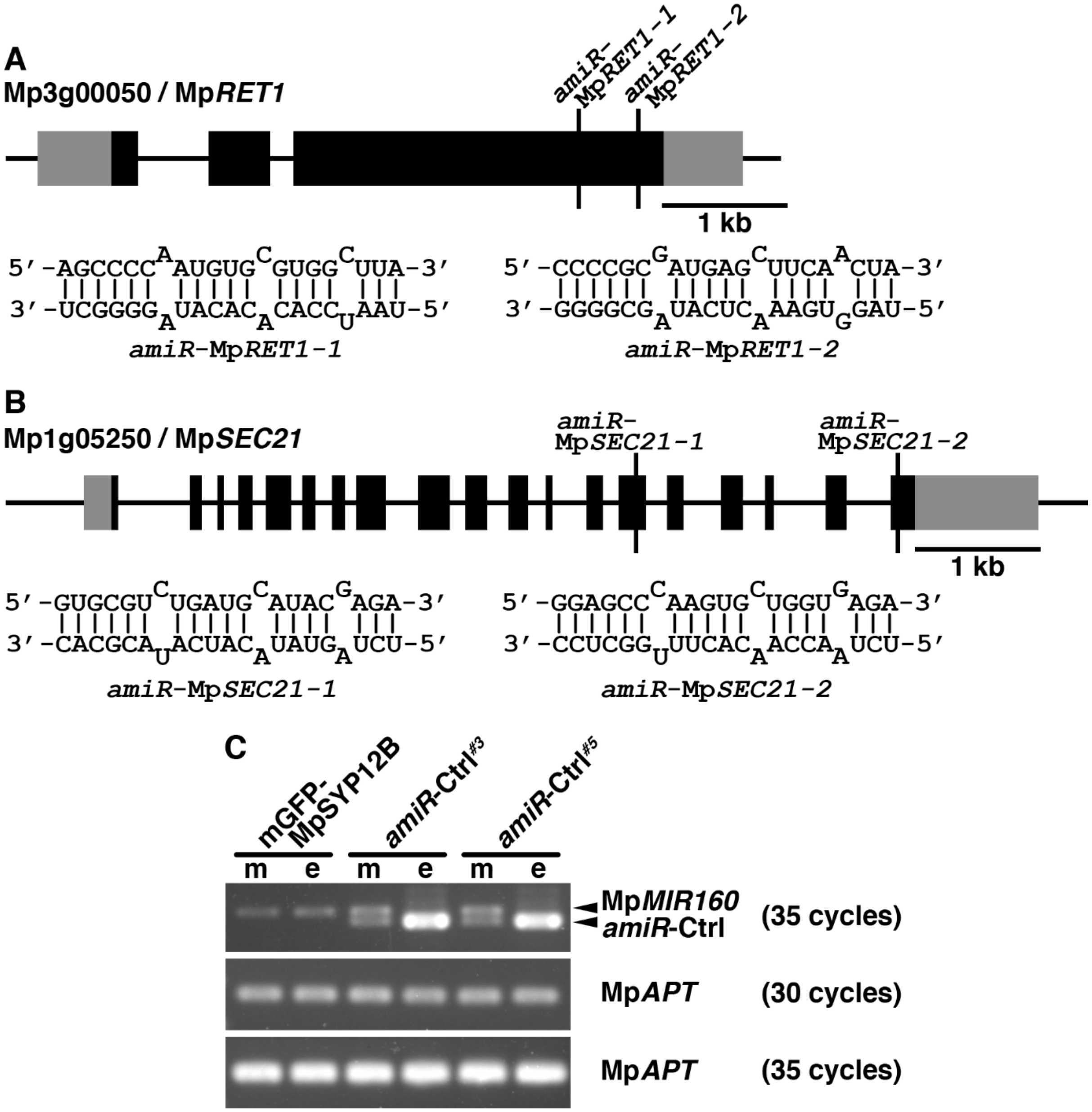

Supplementary Figure 3. The amiRNA target sites in Mp*RET1* and Mp*SEC21*. (A) The Mp*RET1* gene model and *amiR*-Mp*RET1-1* and *amiR*-Mp*RET1-2* target sites. The structures of amiRNA/amiRNA* duplexes for *amiR*-Mp*RET1-1* and *amiR*-Mp*RET1-2* are also shown. **(B)** The Mp*SEC21* gene model and *amiR*-Mp*SEC21-1* and *amiR*-Mp*SEC21-2* target sites. The amiRNA/amiRNA* duplex structures for *amiR*-Mp*SEC21-1* and *amiR*-Mp*SEC21-2* are also shown. Gray and black boxes indicate exons for the UTR and coding sequences, respectively. **(C)** The mRNA expression of a vector control (XVE>>*amiR*-Ctrl). Mp*MIR160* was used as a backbone to design amiRNAs in this study. The target sequence was substituted with *Sma*I and *Hin*dIII sites to construct XVE>>*amiR*-Ctrl. Mp*APT* was used as a loading control. The number of amplification cycles is also shown. m: mock (0.1% (v/v) DMSO); e: 20 μM β-estradiol. XVE>>*amiR*-Ctrl mRNA was weakly expressed under the mock-treated condition and did not affect Mp*RET1* and Mp*SEC21* mRNA expression or thallus growth (Figures 2 and 3 and Supplementary Figures 4 and 5).

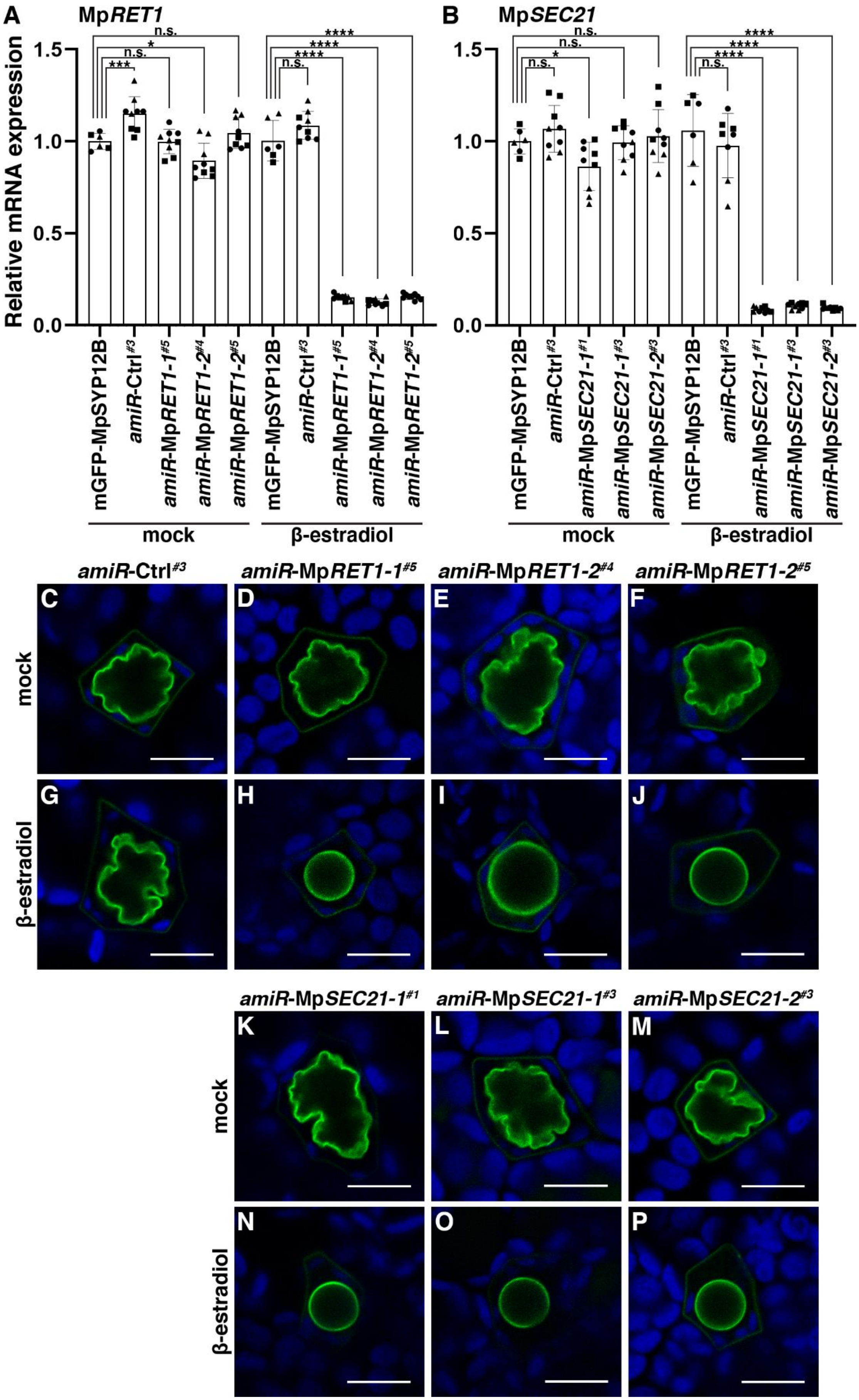
A
Mp*RET1*
B
Mp*SEC21*
Relative mRNA expression
1.5
1.0
0.5
0.0
n.s.
***
*
****
mGFP-MpSYP12B
amiR-Ctrl#3
amiR-Mp*RET1-1*#5
amiR-Mp*RET1-2*#4
amiR-Mp*RET1-2*#5
amiR-Mp*SEC21-1*#1
amiR-Mp*SEC21-1*#3
amiR-Mp*SEC21-2*#3
mock
β-estradiol
C
D
E
F
G
H
I
J
K
L
M
N
O
P

Supplementary Figure 4. Defective COPI function results in abnormally shaped oil bodies. (A) Relative Mp*RET1* mRNA levels in *M. polymorpha* mGFP-MpSYP12B, *amiR*-Ctrl$^{\#3}$, *amiR*-Mp*RET1-1*$^{\#5}$, *amiR*-Mp*RET1-2*$^{\#4}$, and *amiR*-Mp*RET1-2*$^{\#5}$ transgenic plants measured by qRT-PCR. **(B)** Relative Mp*SEC21* mRNA levels in mGFP-MpSYP12B, *amiR*-Ctrl$^{\#3}$, *amiR*-Mp*SEC21-1*$^{\#1}$, *amiR*-Mp*SEC21-1*$^{\#3}$, and *amiR*-Mp*SEC21-2*$^{\#3}$ plants. The same data set in Figure 2 was used for the expression profiles in mGFP-MpSYP12B plants. Mp*APT* was used as an internal reference. Data are presented as the means ± s.d. Significant differences between mGFP-MpSYP12B and each genotype were statistically analyzed by a two-tailed Welch's *t*-test. n.s.: not significant; *$p < 0.05$, ***$p < 0.005$, and ****$p < 0.001$. Three biological replicates were prepared, and experiments were performed two or three times for each gene. Each biological replicate is indicated by a circle (replicate 1), triangle (replicate 2), or square (replicate 3). **(C–P)** Single confocal images of *M. polymorpha* thallus oil body cells of *amiR*-Ctrl$^{\#3}$ **(C, G)**, *amiR*-Mp*RET1-1*$^{\#5}$ **(D, H)**, *amiR*-Mp*RET1-2*$^{\#4}$ **(E, I)**, *amiR*-Mp*RET1-2*$^{\#5}$ **(F, J)**, *amiR*-Mp*SEC21-1*$^{\#1}$ **(K, N)**, *amiR*-Mp*SEC21-1*$^{\#3}$ **(L, O)**, and *amiR*-Mp*SEC21-2*$^{\#3}$ **(M, P)**. Thalli were observed after 48 hr of incubation with 0.1% (v/v) DMSO (mock) **(C–F, K–M)** or 20 µM β-estradiol **(G–J, N–P)**. Green and blue pseudo-colors indicate the fluorescence from mGFP and chlorophyll, respectively. Bars = 10 µm. Representative images are shown among replicates, and the Source data lists the number of observed cells.

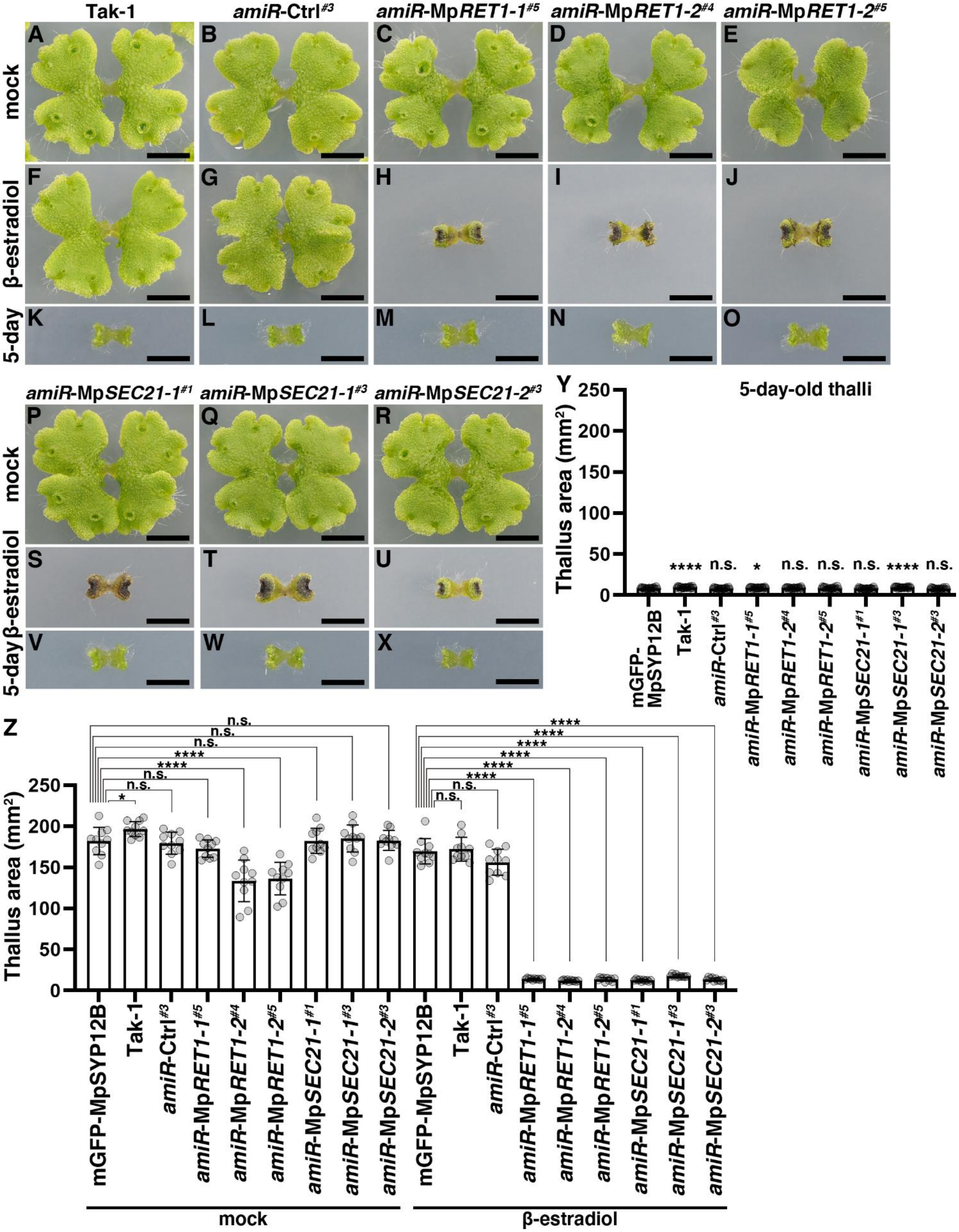

Tak-1
amiR-Ctrl#3
amiR-MpRET1-1#5
amiR-MpRET1-2#4
amiR-MpRET1-2#5
mock
β-estradiol
5-day
amiR-MpSEC21-1#1
amiR-MpSEC21-1#3
amiR-MpSEC21-2#3
5-day-old thalli
Thallus area (mm2)
mGFP-MpSYP12B
n.s.
****
*
mock
β-estradiol

Supplementary Figure 5. Effects of impaired COPI function on thallus growth. (A–X) *M. polymorpha* thalli of Tak-1 **(A, F, K)**, *amiR*-Ctrl$^{\#3}$ **(B, G, L)**, *amiR*-Mp*RET1-1*$^{\#5}$ **(C, H, M)**, *amiR*-Mp*RET1-2*$^{\#4}$ **(D, I, N)**, *amiR*-Mp*RET1-2*$^{\#5}$**(E, J, O)**, *amiR*-Mp*SEC21-1*$^{\#1}$**(P, S, V)**, *amiR*-Mp*SEC21-1*$^{\#3}$**(Q, T, W)**, and *amiR*-Mp*SEC21-2*$^{\#3}$plants **(R, U, X)**. Five-day-old thalli **(K–O, V–X)** were transferred and grown on a medium containing 0.1% (v/v) DMSO (mock) **(A–E, P–R)** or 20 μM β-estradiol **(F–J, S–U)** for seven days. Bars = 5 mm. **(Y, Z)** The thallus area of plants with each genotype at five-days-old **(Y)** and after the mock or β-estradiol treatment **(Z)**. Data are presented as the means ± s.d. Significant differences between mGFP-MpSYP12B and each genotype were statistically analyzed by a two-tailed Welch's *t*-test. n.s.: not significant; $*p < 0.05$, $**p < 0.01$, and $****p < 0.001$. Twenty plants were analyzed for each genotype of five-day-old thalli, and ten plants were analyzed for each genotype of mock- or β-estradiol-treated thalli. The same data set in Figure 3 was used for the mGFP-MpSYP12B plants.

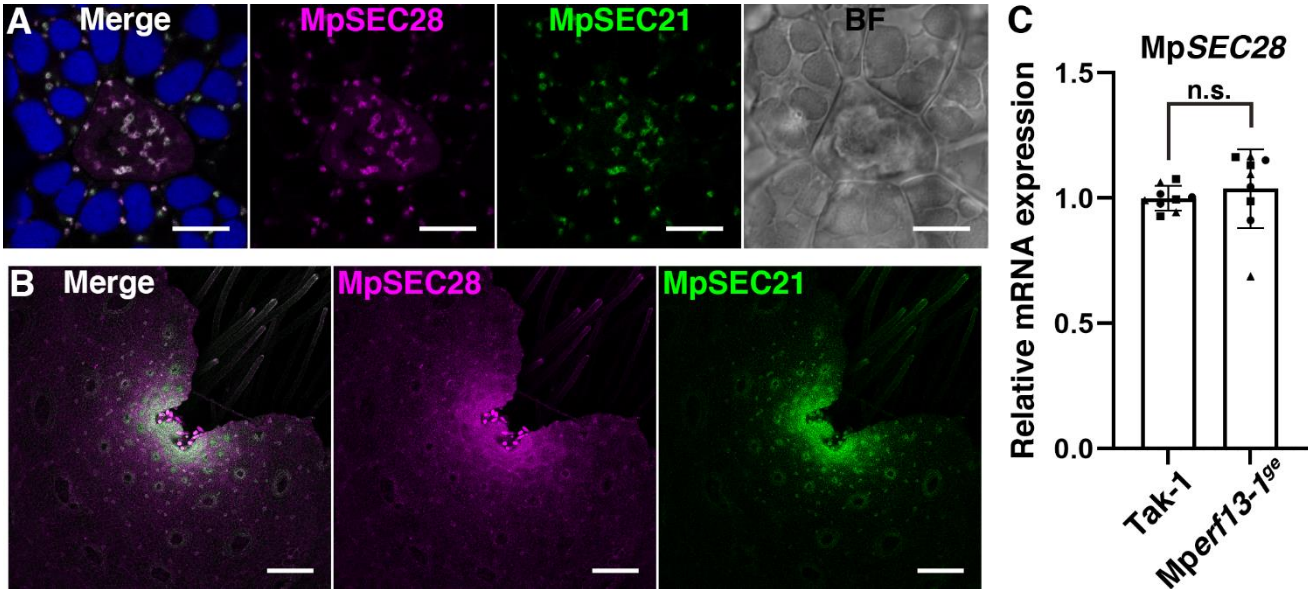

Supplementary Figure 6. Ubiquitous MpSEC28 expression in *M. polymorpha* thalli. (A) Single confocal images of *M. polymorpha* thallus cells expressing MpSEC28-mCherry and MpSEC21-mCitrine driven by their own promoters. BF: bright field. Bars = 10 µm. **(B)** Maximum intensity projection images of the meristematic region of an *M. polymorpha* thallus expressing MpSEC28-mCherry and MpSEC21-mCitrine. Bars = 200 µm. Magenta, green, and blue pseudo-colors indicate fluorescence from mCherry, mCitrine, and chlorophyll, respectively. **(C)** Relative Mp*SEC28* mRNA expression in Tak-1 and Mp*erf13-1*^{ge} measured by qRT-PCR. Mp*APT* was used as an internal reference. Data are presented as the means ± s.d. Significant differences were statistically analyzed between Tak-1 and Mp*erf13-1*^{ge} by a two-tailed Welch's *t*-test. n.s.: not significant. Three biological replicates were prepared, and experiments were performed three times for each gene. Each biological replicate is indicated by a circle (replicate 1), triangle (replicate 2), or square (replicate 3).

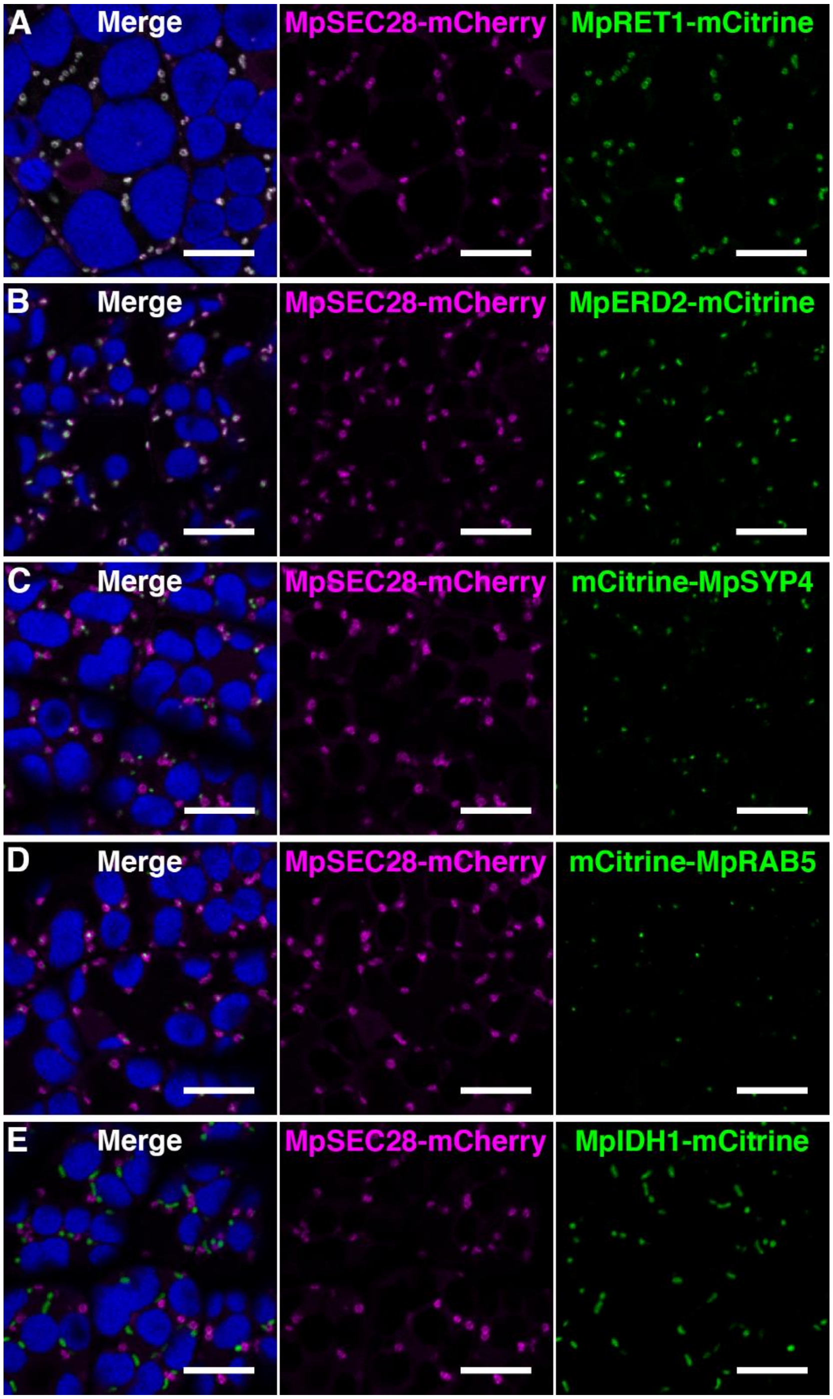

Supplementary Figure 7. MpSEC28-mCherry colocalization with COPI components. (A–E) Single confocal images of *M. polymorpha* thallus cells expressing MpSEC28-mCherry and MpRET1-mCitrine **(A)**, MpERD2-mCitrine **(B)**, mCitrine-MpSYP4 **(C)**, mCitrine-MpRAB5 **(D)**, or MpIDH1-mCitrine **(E)**. Magenta, green, and blue pseudo-colors indicate fluorescence from mCherry, mCitrine, and chlorophyll, respectively. Bars = 10 µm. Figure 4 shows the Pearson correlation coefficients between fluorescence from MpSEC28-mCherry and each organelle marker.

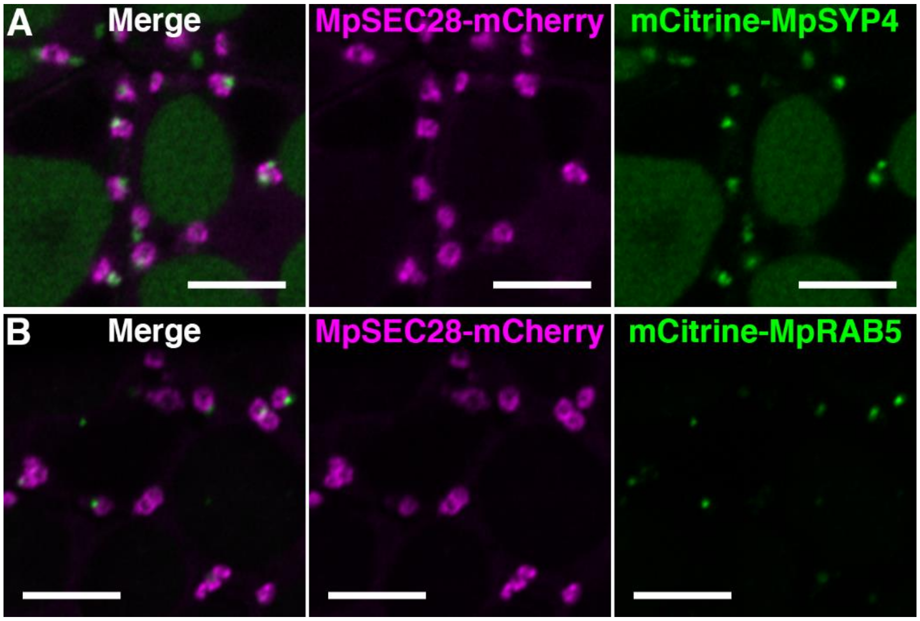

Supplementary Figure 8. High-resolution observation of MpSEC28-mCherry. (A, B) *M. polymorpha* thallus cells expressing MpSEC28-mCherry and mCitrine-MpSYP4 **(A)** or mCitrine-MpRAB5 **(B)** were observed using the Airyscan unit. Magenta and green pseudo-colors indicate fluorescence from mCherry and mCitrine, respectively. Autofluorescence from chlorophyll in chloroplasts was also detected in the channel of mCitrine. Bars = 5 µm.

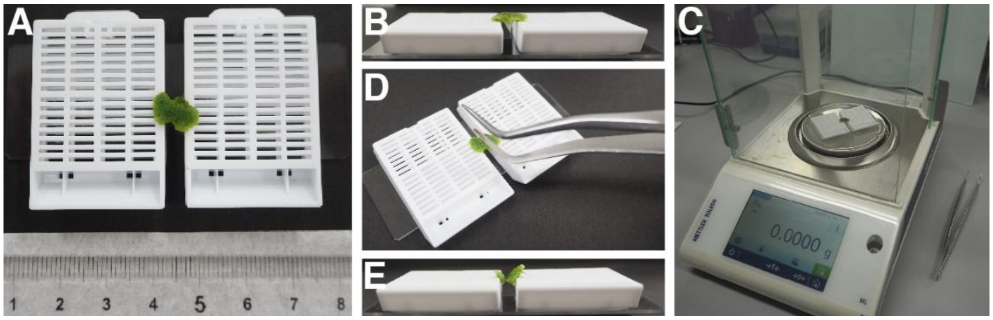

Supplementary Figure 9. Scheme of the chopping assay. (A, B) Twelve-day-old thalli were put on 5 mm gapped blocks. The top view **(A)** and side view **(B)** are shown. **(C, D)** The blocks were placed on an electronic force balance **(C)**, the thalli were pressed using tweezers, and the force that severed the thalli was recorded **(D)**. **(E)** Thallus after the chopping assay.

Supplementary Table 1. Plant strains used in this study.

Experimental Models: Organisms/Strains	Source
Marchantia polymorpha Tak-1	Ishizaki et al., 2008
Marchantia polymorpha Tak-2	Ishizaki et al., 2008
Marchantia polymorpha Mp*sec28-1*	This study
Marchantia polymorpha Mp*sec28-2*^{ge}	This study
Marchantia polymorpha Mp*sec28-3*^{ge}	This study
Marchantia polymorpha mGFP-MpSYP12B	This study
Marchantia polymorpha mGFP-MpSYP12B / XVE>>*amiR*-Ctrl	This study
Marchantia polymorpha mGFP-MpSYP12B / XVE>>*amiR*-Mp*RET1-1*	This study
Marchantia polymorpha mGFP-MpSYP12B / XVE>>*amiR*-Mp*RET1-2*	This study
Marchantia polymorpha mGFP-MpSYP12B / XVE>>*amiR*-Mp*SEC21-1*	This study
Marchantia polymorpha mGFP-MpSYP12B / XVE>>*amiR*-Mp*SEC21-2*	This study
Marchantia polymorpha MpSEC28-mCherry / MpSEC21-mCitrine	This study
Marchantia polymorpha MpSEC28-mCherry / mCitrine-MpGOS11	This study
Marchantia polymorpha MpSEC28-mCherry / MpRET1-mCitrine	This study
Marchantia polymorpha MpSEC28-mCherry / MpERD2-mCitrine	This study
Marchantia polymorpha MpSEC28-mCherry / mCitrine-MpSYP4	This study
Marchantia polymorpha MpSEC28-mCherry / mCitrine-MpRAB5	This study
Marchantia polymorpha MpSEC28-mCherry / MpIDH1-mCitrine	This study
Marchantia polymorpha $_{pro}$Mp*SYP12B:*sec-mRFP	Kanazawa et al., 2020
Marchantia polymorpha Mp*sec28-1* / $_{pro}$Mp*SYP12B:*sec-mRFP	This study
Marchantia polymorpha Mp*sec28-2*^{ge} / $_{pro}$Mp*SYP12B:*sec-mRFP	This study
Marchantia polymorpha Mp*sec28-3*^{ge} / $_{pro}$Mp*SYP12B:*sec-mRFP	This study
Marchantia polymorpha Mp*sec28-1* / mGFP-MpSYP12B	This study

Supplementary Table 2. Oligonucleotides used in this study.

Genes	5′ → 3′	Size (kb)
**Entry clone**		
Mp*SEC28* (CDS)	CACCATGAGTGGAGGACCAGATCCCCTG<br>AGCTCTCTCGAAAGGTTGAACAGC	0.9
*pro*Mp*SEC28*-*Not*I	GCAGGCTCCGCGGCCGCTTGAGTTATGGAGGTTTTTAGTGG<br>GTGAAGGGGGCGGCCGTTTCTAAGCTCCAACTGTTGGAATTCG	5.6
mCherry-*Asc*I	AAGGGTGGGCGCGGCGGCGGCAGCGGCGGCGGCAGCGGCATGGTG AGCAAGGGCGAGGAGG<br>AGCTGGGTCGGCGCGTTACTTGTACAGCTCGTCCATGCC	0.8
mGFP-*Sma*I (MpSYP12B)	GCCCCCTTCACCCCCATGGTGAGCAAGGGCGAGGAG<br>CATGCCGCTGCCCCCCTTGTACAGCTCGTCCATGCC	0.7
Mp*SEC21* (CDS+intron)	CACCATGGCTCAACTCCAGCCCGTCG<br>TGCACTCCCTATGATCTCGTGAATC	6.4
*pro*Mp*SEC21*-*Not*I	GCAGGCTCCGCGGCCCAAGATCATTCGATTTATTCGACAAATC<br>GTGAAGGGGGCGGCCCGCAGCAAACTCAGATTGCCTGCCTGC	2.9
Mp*RET1* (CDS)	CACCATGCTGACTAAGTTCGAGACCAAG<br>CCGCACTTGGGTAGGAGAGCAGAGC	3.7
mCitrine-*Asc*I	AAGGGTGGGCGCGGCGGCGGCAGCGGCGGCGGCAGCGGCATGGTG AGCAAGGGCGAGGAGCTG<br>AGCTGGGTCGGCGCGTTACTTGTACAGCTCGTCCATGC	0.8
Mp*ERD2*	CACCATGAATATCTTCAGATTGGCTGG<br>GGCCGGTAGATTGAGCCTCTC	0.7
**Destination vector**		
*pro*Mp*SYP2*-*Hind*III	GGCCAGTGCCAAGCTCACGAGCGAGTGAGACACCAGAGGAG<br>TTTGTACAAACTTGTCCTCCTGCTTCGTGGTAAATCCTCTTC	4.3
mCitrine-*Sac*I	gtggttgataacagcATGGTGAGCAAGGGCGAGGAGC<br>gatcggggaaattcgTCACTTGTACAGCTCGTCCATGC	0.7
**Genome edit**		
gRNA1 MpSEC28	ctcgGAGAGGAGAAGGTGCCGCAA<br>aaacTTGCGGCACCTTCTCCTCTC	-
gRNA2 MpSEC28	ctcgTTGGAGCTTAGAAACATGAG<br>aaacCTCATGTTTCTAAGCTCCAA	-

gRNA3 MpSEC28	ctcgTAAGTAGATCAGGTGTGATT<br>aaacAATCACACCTGATCTACTTA	-
gRNA4 MpSEC28	ctcgGAATCCGACACATTCGGACA<br>aaacTGTCCGAATGTGTCGGATTC	-
amiRNA		
amiR-Mp*RET1-1*	CACCGCACCTCCTCTCTCCGACTGCAGCCCGTTTCGAGATCCGAGGACTTGCTCGACGCGACTAATTGGGGAGGCCAGACTGCACTTAAGCCACGCACATTGGGGCTACTGAGGAGCTCCTCAGAGACCTTGACAGGCTCCGTAG<br>TAAGTAAATCTATCAAACATCAGTGAGGTGTGCACCCAAGACAGAAAGCTAGTCGCACCTTGCGGGAGGTAGCGACTTCCTCCTAAtCCACaCACATaGGGGCTGCTACGGAGCCTGTCAAGGTCTCTGAGGAGCTCCTCAGTAG	-
amiR-Mp*RET1-2*	CACCGCACCTCCTCTCTCCGACTGCAGCCCGTTTCGAGATCCGAGGACTTGCTCGACGCGACTAATTGGGGAGGCCAGACTGCACTTAGTTGAAGCTCATCGCGGGGACTGAGGAGCTCCTCAGAGACCTTGACAGGCTCCGTAG<br>TAAGTAAATCTATCAAACATCAGTGAGGTGTGCACCCAAGACAGAAAGCTAGTCGCACCTTGCGGGAGGTAGCGACTTCCTCCTAGgTGAAaCTCATaGCGGGGGCTACGGAGCCTGTCAAGGTCTCTGAGGAGCTCCTCAGTCC	-
amiR-Mp*SEC21-1*	CACCGCACCTCCTCTCTCCGACTGCAGCCCGTTTCGAGATCCGAGGACTTGCTCGACGCGACTAATTGGGGAGGCCAGACTGCACTTCTCGTATGCATCAGACGCACACTGAGGAGCTCCTCAGAGACCTTGACAGGCTCCGTAG<br>TAAGTAAATCTATCAAACATCAGTGAGGTGTGCACCCAAGACAGAAAGCTAGTCGCACCTTGCGGGAGGTAGCGACTTCCTCCTCTaGTATaCATCAtACGCACGCTACGGAGCCTGTCAAGGTCTCTGAGGAGCTCCTCAGTGT	-
amiR-Mp*SEC21-2*	CACCGCACCTCCTCTCTCCGACTGCAGCCCGTTTCGAGATCCGAGGACTTGCTCGACGCGACTAATTGGGGAGGCCAGACTGCACTTCTCACCAGCACTTGGGCTCCACTGAGGAGCTCCTCAGAGACCTTGACAGGCTCCGTAG<br>TAAGTAAATCTATCAAACATCAGTGAGGTGTGCACCCAAGACAGAAAGCTAGTCGCACCTTGCGGGAGGTAGCGACTTCCTCCTCTaACCAaCACTTtGGCTCCGCTACGGAGCCTGTCAAGGTCTCTGAGGAGCTCCTCAGTGG	-
amiR-Ctrl	CACCGCACCTCCTCTCTCCGACTGCAGCCCGTTTCGAGATCCGAGGACTTGCTCGACGCGACTAATTGGGGAGGCCAGACTGCACTcccgggACTGAGGAGCTCCTCAGAGACCTTGACAGGCTCCGTAG<br>TAAGTAAATCTATCAAACATCAGTGAGGTGTGCACCCAAGACAGAAAGCTAGTCGCACCTTGCGGGAGGTAGCGACTTCCTCCaagcttGCTACGGAGCCTGTCAAGGTCTCTGAGGAGCTCCTCAGTcc	-
Mp*MIR160*	CACCGCACCTCCTCTCTCCGACTGCAG	250 bp

	TAAGTAAATCTATCAAACATCAG	
Genotyping PCR		
Mp*sec28-1*	GAATAGCGTTCCGTCGTCTAAACG	WT: 0.5 kb, Mp*sec28-1*: 3.2 kb
	GCTCCAACTGTTGGAATTCGCCCG	
Mp*sec28-2*^{ge} Mp*sec28-3*^{ge}	GAATAGCGTTCCGTCGTCTAAACG	WT: 1.6 kb, Mp*sec28-2*^{ge}: 1.1 kb, Mp*sec28-3*^{ge}: 0.8 kb
	GCTGAGCTGCTAATTTCATCGATCAC	
RT-PCR		
Mp*MIR160*	CACCGCACCTCCTCTCTCCGACTGCAG	Mp*MIR160*: 250 bp, *amiR*-Ctrl: 220 bp
	TAAGTAAATCTATCAAACATCAG	
Mp*APT*	CGAAAGCCCAAGAAGCTACC	146 bp
	GTACCCCCGGTTGCAATAAG	
qRT-PCR		
Mp*RET1*	GACAGACGTACACTCCCATC	135 bp
	CAGAAGCATCTGCTCCGACG	
Mp*SEC21*	GCTCCTCGACGATGTAAGTGT	243 bp
	AGCTGGTACTCGTCCTCATAAC	
Mp*SEC28*	GTTTCAGGCTGCGATCAACG	88 bp
	GTAGGAGCGATACACGAGGC	
Mp*APT*	CGAAAGCCCAAGAAGCTACC	146 bp
	GTACCCCCGGTTGCAATAAG	

Supplementary Table 3. Plasmid DNA used in this study.

Plasmid DNA	Source	Identifier
pENTR Genomic mGFP-MpSYP12B	This study	
pENTR $_{pro}$Mp*SEC28:*MpSEC28-mCherry	This study	
pENTR $_{pro}$Mp*SEC21:*MpSEC21	This study	
pENTR MpRET1 (CDS)-mCitrine	This study	
pENTR MpERD2 (CDS)	This study	
pENTR *amiR*-Ctrl	This study	
pENTR *amiR*-Mp*RET1-1*	This study	
pENTR *amiR*-Mp*RET1-2*	This study	
pENTR *amiR*-Mp*SEC21-1*	This study	
pENTR *amiR*-Mp*SEC21-2*	This study	
pMpGE_En04	Koide et al., 2020	
pMpGE_En04 gRNA1_MpSEC28	This study	
pMpGE_En04 gRNA2_MpSEC28	This study	
pBC-GE14	Koide et al., 2020	
pBC-GE14 gRNA3_MpSEC28	This study	
pBC-GE14 gRNA4_MpSEC28	This study	
pMpGE_En04 gRNA1-3_Mp*sec28-2*^{ge}	This study	
pMpGE_En04 gRNA2-4_Mp*sec28-3*^{ge}	This study	
pMpGE011	Sugano et al., 2018	GenBank: LC090757
pMpGE011 gRNA1-3_Mp*sec28-2*^{ge}	This study	
pMpGE011 gRNA2-4_Mp*sec28-3*^{ge}	This study	
pMpGWB368	Unpublished*	
pMpGWB368 *amiR*-Mp*RET1-1*	This study	
pMpGWB368 *amiR*-Mp*RET1-2*	This study	
pMpGWB368 *amiR*-Mp*SEC21-1*	This study	
pMpGWB368 *amiR*-Mp*SEC21-2*	This study	
pMpGWB368 *amiR*-Ctrl	This study	
pMpGWB101	Ishizaki et al., 2015	GenBank: LC057443
pMpGWB301	Ishizaki et al., 2015	GenBank: LC057517
pMpGWB301 GW-mCitrine	This study	
pMpGWB301 $_{pro}$Mp*SYP2:*GW	This study	

pMpGWB301 *pro*Mp*SYP2:*GW-mCitrine	Norizuki et al., 2022
pMpGWB101 Genomic mGFP-MpSYP12B	This study
pMpGWB301 Genomic mGFP-MpSYP12B	This study
pMpGWB101 *pro*Mp*SEC28:*MpSEC28-mCherry	This study
pMpGWB301 *pro*Mp*SEC21:*MpSEC21-mCitrine	This study
pMpGWB301 *pro*Mp*SYP2:*mCitrine-MpGOS11	Minamino et al., 2017
pMpGWB301 *pro*Mp*SYP2:*MpRET1-mCitrine	This study
pMpGWB301 *pro*Mp*SYP2:*MpERD2-mCitrine	This study
pMpGWB301 Genomic mCitrine-MpSYP4	Kanazawa et al., 2020
pMpGWB301 Genomic mCitrine-MpRAB5	Minamino et al., 2017
pMpGWB301 *pro*Mp*SYP2:*MpIDH1-mCitrine	Norizuki et al., 2022
pMpGWB101 *pro*Mp*SYP12B:*sec-mRFP	This study
pMpGWB301 *pro*Mp*SYP12B:*sec-mRFP	Kanazawa et al., 2020

*Details will be published elsewhere. The β-estradiol inducible cassette was described in Ishida et al., 2022 and Furuya et al., 2022.

Supplementary Table 4. Gene accession number. The names of the *Marchantia polymorpha* genes and proteins follow those of Bowman et al. (2016).

Gene symbol	Ver 3.1	Ver 5.1
Mp*SEC28*	Mapoly0002s0056	Mp1g28220
Mp*SEC21*	Mapoly0005s0083	Mp1g05250
Mp*RET1*	Mapoly0007s0005	Mp3g00050
Mp*MIR160*	Mapoly0002s0211	Mp1g26670
Mp*APT*	Mapoly0100s0027	Mp3g25140
Mp*SYP12B*	Mapoly0101s0013	Mp4g20670
Mp*SYP2*	Mapoly0187s0013	Mp8g15260
Mp*GOS11*	Mapoly0016s0161	Mp6g11210
Mp*ERD2*	Mapoly0044s0129	Mp4g03440
Mp*SYP4*	Mapoly0042s0041	Mp2g14140
Mp*RAB5*	Mapoly0036s0134	Mp1g08940
Mp*IDH1*	Mapoly0029s0048	Mp1g01980
Mp*ERF13*	Mapoly0060s0052	Mp6g08690

References in Supplementary Data

Bowman, J. L., Araki, T., Arteaga-Vazquez, M. A., Berger, F., Dolan, L., Haseloff, J., et al. (2016). The naming of names: guidelines for gene nomenclature in Marchantia. *Plant Cell Physiol*. 57, 257–261. doi: 10.1093/pcp/pcv193

Furuya, T., Nishihama, R., Ishizaki, K., Kohchi, T., Fukuda, H., and Kondo, Y. (2022) A glycogen synthase kinase 3-like kinase MpGSK regulates cell differentiation in *Marchantia polymorpha*. *Plant Biotech*. 39: 65-72. doi: 10.5511/plantbiotechnology.21.1219a

Ishida, S., Suzuki, H., Iwaki, A., Kawamura, S., Yamaoka, S., Kojima, M., et al. (2022) Diminished auxin signaling triggers cellular reprogramming by inducing a regeneration factor in the liverwort *Marchantia polymorpha*. *Plant Cell Physiol*. 63:384-400. doi: 10.1093/pcp/pcac004

Ishizaki, K., Chiyoda, S., Yamato, K. T., and Kohchi, T. (2008). Agrobacterium-mediated transformation of the haploid liverwort *Marchantia polymorpha* L., an emerging model for plant biology. *Plant Cell Physiol*. 49, 1084–1091. doi: 10.1093/pcp/pcn085

Ishizaki, K., Nishihama, R., Ueda, M., Inoue, K., Ishida, S., Nishimura, Y., et al. (2015). Development of gateway binary vector series with four different selection markers for the liverwort *Marchantia polymorpha*. *PLoS One* 10:e0138876. doi: 10.1371/journal.pone.0138876

Kanazawa, T., Morinaka, H., Ebine, K., Shimada, T. L., Ishida, S., Minamino, N., et al. (2020) The liverwort oil body is formed by redirection of the secretory pathway. *Nat Commun*. 11:6152. doi: 10.1038/s41467-020-19978-1

Koide, E., Suetsugu, N., Iwano, M., Gotoh, E., Nomura, Y., Stolze, S.C., et al. (2020) Regulation of photosynthetic carbohydrate metabolism by a Raf-like kinase in the liverwort *Marchantia polymorpha*. *Plant Cell Physiol*. 61:631-643. doi: 10.1093/pcp/pcz232

Minamino, N., Kanazawa, T., Nishihama, R., Yamato, K. T., Ishizaki, K., Kohchi, T., et al. (2017) Dynamic reorganization of the endomembrane system during spermatogenesis in *Marchantia polymorpha*. *J Plant Res*. 130:433–441. doi: 10.1007/s10265-017-0909-5

Norizuki, T., Minamino, N., Sato, M., Tsukaya, H., and Ueda, T. (2022) Dynamic rearrangement and autophagic degradation of mitochondria during spermiogenesis in the liverwort *Marchantia polymorpha*. *Cell Rep.* 39:110975. doi: 10.1016/j.celrep.2022.110975

Sugano, S. S., Nishihama, R., Shirakawa, M., Takagi, J., Matsuda, Y., Ishida, S., et al. (2018). Efficient CRISPR/Cas9-based genome editing and its application to conditional genetic analysis in *Marchantia polymorpha*. *PLoS One* 13:e0205117. doi: 10.1371/journal.pone.0205117
